# Ti_3_C_2_T_*x*_ MXene Nanosheets: Bridging High-Performance Energy Storage and Comprehensive In Vivo Biocompatibility Assessment

**DOI:** 10.1021/acsami.6c05337

**Published:** 2026-06-15

**Authors:** Tanveer Ali, Ali Shan, Mirza Mahmood Baig, He Xu, Zhihao Zhao, Hou Yuexue, Sooman Lim, Seung Goo Lee, Lin Zhang

**Affiliations:** † Institute of Integrative Medicine, 36674 Dalian Medical University, Dalian 0411, China; ‡ Faculty of Eastern Medicine and Natural Sciences, Ziauddin University, Karachi 74600, Pakistan; § Graduate School of Flexible and Printable Electronics, LANL, JBNU Engineering Institute, 26714 Jeonbuk National University, Jeonbuk 54896, Republic of Korea; ∥ Department of Chemistry, 35029 University of Ulsan, Ulsan 44610, Republic of Korea

**Keywords:** asymmetric supercapacitor, biosafety, biocompatibility, in vivo toxicity, MXene

## Abstract

Owing to advancements in implantable bioelectronic devices, there has been an increase in demand for biocompatible energy sources with long-term electrochemical and mechanical stability. In this study, we present the fabrication of a flexible asymmetric supercapacitor (MXene//AC) based on two-dimensional Ti_3_C_2_T*_x_* MXene nanosheets. The supercapacitor demonstrates excellent electrochemical performance with an areal capacitance of 66.43 mF cm^–2^, energy density of 13.2 Wh kg^–1^, and a high power density of 2300 W kg^–1^. The supercapacitor retained 95% capacitance after 5000 charge–discharge cycles and displayed negligible performance degradation under various bending angles, highlighting its mechanical suitability for wearable electronics. Density functional theory (DFT) analysis revealed that the metallic Ti–C backbone of Ti3C2T*_x_* MXene and its O/F terminations work synergistically to enable rapid electron transport and reversible proton-coupled surface redox, supporting predominantly surface-controlled charge storage with a significant pseudocapacitive contribution. To complement device-level studies, we assessed the in vivo safety profile and antioxidant potential of Ti_3_C_2_T*_x_* MXene nanosheets in Sprague–Dawley (SD) rats through acute dermal, subchronic oral, and subchronic intraperitoneal toxicity evaluations. Acute dermal exposure up to 100 mg kg^–1^ caused mild skin responses without necrosis, while subchronic administration for 28 days revealed no considerable abnormalities in biochemical parameters (alanine aminotransferase, aspartate aminotransferase, blood urea nitrogen, creatinine), inflammatory markers (IL-1β, IL-6), or oxidative stress biomarkers (malondialdehyde, glutathione). Histopathological evaluations confirmed the absence of structural damage or inflammation in vital organs. Additionally, MXene nanosheets demonstrated antioxidant activity by scavenging 2,2-azino-bis­(3-ethyl)­benzothiazoline-6-sulfonic acid free radicals in a dose-dependent manner, highlighting their potential to reduce oxidative stress in biomedical applications. Overall, this dual-focused study demonstrates that MXene nanosheets are not only highly effective for developing flexible, stable asymmetric supercapacitors but also exhibit favorable in vivo biocompatibility and antioxidant properties at the tested doses. These findings emphasize the potential of MXene-based materials as next-generation, fully biocompatible energy storage devices for advanced implantable bioelectronic systems.

## Graphical abstract

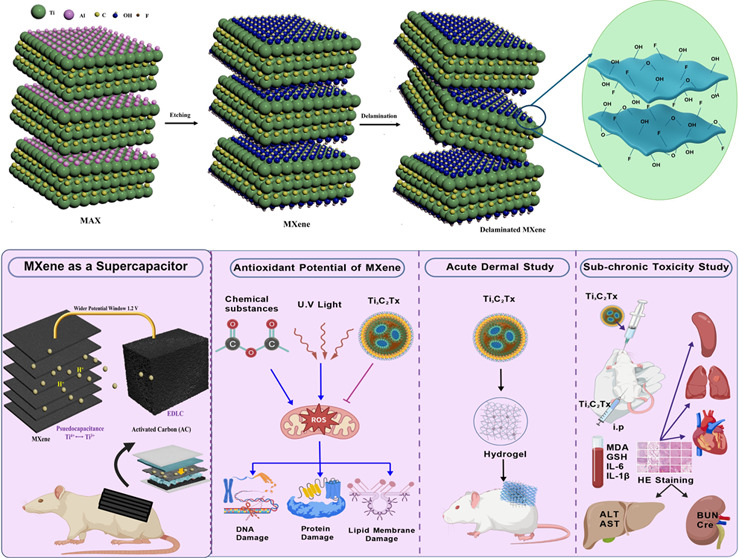

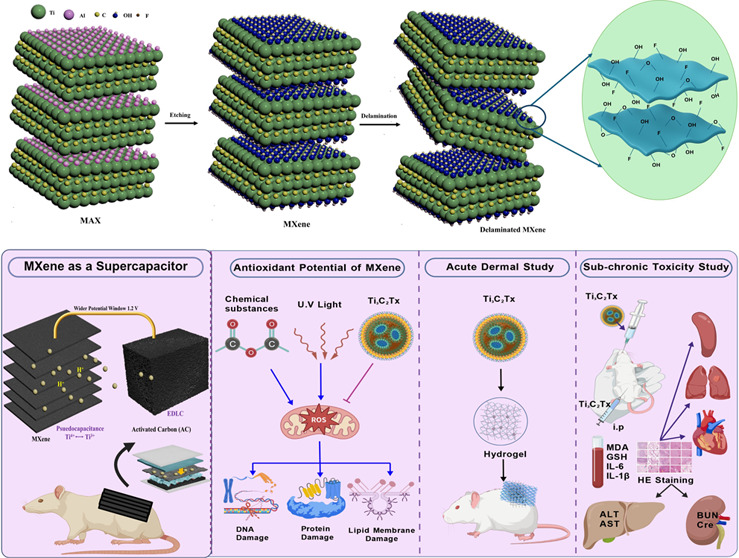

## 1 Introduction

Due to a global demand for sustainable and reliable sources, energy storage technologies are emerging as vital components in modern society. Conventional energy storage devices, such as batteries and fuel cells, provide high energy density; however, they often suffer from low power density, along with slow charge and discharge rates, thereby limiting their use in applications requiring rapid energy delivery. On the other hand, capacitors offer high power density but relatively low energy storage capacity. Supercapacitors, bridging the gap between batteries and capacitors, offer a combination of high energy and power density, rapid charge and discharge rates, and long cycling stability, making them highly attractive for next-generation energy storage applications.^1,2^

Supercapacitor performance heavily depends on the electrode materials. Metal oxides provide high theoretical capacitance but suffer from high charge-transfer resistance, which limits ion transport and redox activity. Carbon-based materials, including graphene and reduced graphene oxide, offer excellent conductivity and chemical stability; however, their energy storage primarily relies on physical charge accumulation, limiting the number of active sites. Hybrid materials, combining carbon-based frameworks with metal oxides or other functional nanostructures, have been investigated to address the limitations posed by the aforementioned materials, thereby offering improved conductivity, more electroactive sites, and enhanced energy storage efficiency.^3,4^

Among emerging electrode materials, two-dimensional (2D) MXenes, particularly Ti_3_C_2_T_*x*_, have demonstrated exceptional potential for high-performance energy-storage applications. Since their discovery in 2011, MXenes have attracted considerable attention owing to their ultrathin layered morphology, high specific surface area, metallic electrical conductivity, tunable surface chemistry, and intrinsic hydrophilicity.^5,6^

Compared with other transition-metal (TM)-based MXenes such as Ti_2_CT_*x*_, V_2_CT_*x*_, and Mo-based MXenes, Ti_3_C_2_T_*x*_ generally exhibits higher electrical conductivity, superior structural stability, scalable synthesis, and more efficient charge transport due to its conductive Ti–C metallic backbone. In addition, the layered Ti_3_C_2_ structure provides enhanced mechanical robustness and structural integrity during repeated ion intercalation/deintercalation processes, making it highly suitable for flexible and wearable energy-storage systems.^7^ The rich surface terminations of Ti_3_C_2_T_*x*_, including −O, −OH, and −F groups, facilitate rapid ion transport, improve electrolyte wettability, and enhance electrochemical activity, thereby enabling high capacitance, fast charge/discharge kinetics, and excellent mechanical stability under bending or stretching conditions.^8,9^

Furthermore, Ti_3_C_2_T_*x*_ MXene has shown promising biocompatibility and favorable interactions with biological environments, making it an attractive candidate for implantable and wearable bioelectronic devices positioned near the human body.^10^ The electrochemical and biological properties of MXenes are strongly influenced by the etching method and the resulting surface functional groups. Different etching approaches, including direct HF etching, LiF/HCl-assisted etching, and fluorine-free molten salt routes, can significantly alter defect density, oxidation level, interlayer spacing, conductivity, and surface terminations (−O, −OH, and −F), thereby affecting ion transport, wettability, pseudocapacitive behavior, oxidation stability, and interfacial interactions.^11,12^

In particular, oxygen- and hydroxyl-terminated MXenes generally exhibit enhanced electrolyte accessibility, improved hydrophilicity, and superior pseudocapacitive charge-storage behavior due to their active participation in reversible surface redox reactions. In contrast, excessive fluorine terminations are often considered less favorable because they are relatively electrochemically inactive and may hinder ion diffusion or reduce conductivity. Nevertheless, moderate fluorine content in HF-etched Ti_3_C_2_T_*x*_ can contribute to colloidal stability and hydrophilic surface characteristics. Surface chemistry also plays a crucial role in determining the biocompatibility, biomolecular interactions, oxidative stress response, and antioxidant behavior of MXene-based materials.^13,14^

In this study, Ti_3_C_2_T_*x*_ MXene nanosheets were synthesized through selective HF etching followed by repeated washing and freeze-drying treatment. The repeated washing process helped remove residual acidic species and minimize excessive fluorine-related adverse effects, while the freeze-drying process preserved the layered morphology, enlarged interlayer spacing, and hydrophilic surface chemistry of the MXene nanosheets. Moreover, freeze-drying minimized irreversible restacking of MXene layers, thereby facilitating efficient ion transport and electrochemical accessibility. These structural and surface characteristics collectively contributed to the excellent electrochemical performance, mechanical flexibility, antioxidant activity, and favorable biocompatibility demonstrated in the present work.

Ti_3_C_2_T_*x*_ MXene are highly promising for a broad range of biomedical applications owing to their large surface area, hydrophilicity, conductivity, 2D structure, and tunable particle size. These characteristics allow for their successful integration into hybrid nanotechnology and nanocomposites and improve oxidative stability, target specificity, biodegradability, and physiological biocompatibility.^15^ However, the clinical translation of MXene requires rigorous safety evaluation. Similar 2D materials, such as graphene and TM dichalcogenides (TMDs), are scrutinized due to their unpredictable biocompatibility and potential chronic toxicity.^16^ Preliminary in vitro studies indicate that MXene cytotoxicity depends on lateral size, surface functionalization, and concentration. For example, Ti_3_C_2_T_*x*_ nanosheets smaller than 100 nm induce oxidative stress in human keratinocyte cells at high concentrations (>100 μg/mL), whereas larger sheets (>100 nm) have moderate effects.^17^

In vivo data on MXene toxicity remains limited and occasionally inconsistent. Rozmysłowska-Wojciechowska et al. reported no considerable inflammation in mice following intravenous injection of MXene,^18^ whereas other studies observed dose-dependent toxicity in zebrafish models. Essraa A. Hussein et al. found that Au/MXene and Au/Fe_3_O_4_/MXene nanocomposites exhibited safer profiles in zebrafish embryos than pure MXene.^19^ Other metal-based nanocomposites demonstrated excellent anticancer photothermal therapy efficacy with lower in vivo toxicity than pristine MXene.^20^ Nasrallah et al. evaluated the acute toxicity of Ti_3_C_2_T_*x*_ in zebrafish embryos,^21^ and Sui et al. studied MXene distribution and organ deposition.^22^ Alhussain et al. investigated embryonic toxicity in chicken embryos.^23^ Recently, photothermal therapy studies using MXene under near-infrared radiation further emphasize the need for rigorous in vivo toxicity testing in mammalian models.^24^

These variations illustrate the need for systematic, route-specific toxicity evaluations to establish safe exposure levels. For biomedical applications, careful design of MXene composition, size, and surface functionalization, together with controlled drug delivery strategies, is essential for optimizing MXene therapeutic efficacy.^25^ Currently, comprehensive in vivo studies have not assessed MXene toxicity across multiple administration routes, leaving a critical gap, particularly for potential oral, i.e., gastrointestinal therapeutics, or intraperitoneal, i.e., localized drug delivery, applications.

This study addresses these research gaps by assessing the subchronic toxicological effects of MXene nanoparticles in Sprague–Dawley (SD) rats via oral and intraperitoneal routes, using histopathological, biochemical, and oxidative stress end points. Our findings, showing no significant toxicity at the tested doses, identify MXenes as potentially safe biomaterials and support their integration into medical devices and therapies. Despite the promise of MXene-based biomaterials, challenges such as long-term safety, potential neurotoxicity, and production scalability must be addressed.

To address material and biomedical limitations, we fabricated a flexible asymmetric MXene//AC supercapacitor that exhibited an areal capacitance of 66.43 mF cm^–2^, an energy density of 13.2 Wh kg^–1^, and a power density of 2300 W kg^–1^. The device retained 95% of its capacitance after 5000 cycles and showed negligible performance degradation during repeated bending or attachment to the human hand, demonstrating its suitability for wearable electronics. A combination of excellent electrochemical performance and comprehensive in vivo safety evaluation is foundational for advancing MXene as multifunctional materials for energy storage and biomedical applications, highlighting their potential in wearable electronics, implantable devices, and other emerging technologies.

## 2 Experimental Section

### 2.1 Synthesis of Ti_3_C_2_T_*x*_

The process also helped maintain enlarged interlayer spacing, hydrophilic surface terminations, Ti_3_C_2_T_*x*_ MXene was synthesized by selectively etching the Al layer from Ti_3_AlC_2_ MAX phase using hydrofluoric acid (HF), following the method reported by Naguib et al.^26^ Briefly, 1 g of Ti_3_AlC_2_ powder was gradually added to 15 mL of concentrated HF solution (48%, Adams Beta) under continuous stirring at room temperature and maintained for 48 h to ensure complete etching of the Al layers. After the etching process, the reaction mixture was diluted with approximately 500 mL of deionized (DI) water and centrifuged at 5000 rpm for 10 min to remove acidic byproducts and residual reaction species. The obtained sediment was repeatedly washed with DI water through multiple centrifugation cycles until the supernatant reached a near-neutral pH (∼6.8), indicating effective removal of residual HF and excessive fluorine-containing species. The washed Ti_3_C_2_T_*x*_ sediment was subsequently collected and freeze-dried to preserve the layered two-dimensional morphology and minimize irreversible restacking of MXene nanosheets. The freeze-drying and electrochemically active surface chemistry while reducing excessive fluorine-related adverse effects. Finally, the dried MXene powder was stored at −4 °C for further electrochemical and biological investigations.

### 2.2 Carbon Cloth Treatment

Commercial carbon cloth was used as a substrate. However, the substrate required initial treatments for purification and activation. Therefore, the carbon cloth was treated with 2 M HCl for 8 h at room temperature under constant stirring. Following this, the carbon cloth was treated with 2 M NaOH to obtain a hydrophilic substrate that ensures proper electrode interaction with the aqueous electrolyte. Finally, the electrode was washed with ethanol and distilled water to prepare the substrate for use.

### 2.3 Electrode Fabrication

Electrode fabrication was initiated by preparing an MXene slurry. First, 30 mg of MXene powder was added to 1 mL of 2% Nafion binder solution and sonicated for 2 h under constant water flow to maintain the MXene suspension at room temperature. Afterward, the slurry was drop-cast onto 1 cm^2^ × 1 cm^2^ carbon cloth and dried at 60 °C in a gas oven overnight to obtain the final electrode for application.

### 2.4 Electrochemical Characterization

An electrochemical workstation (WisEIS–1200 Premium) was used to perform CV, GCD, and EIS analyses of MXene. Electrochemical measurements were performed using 3 M H_2_SO_4_ as the electrolyte, Hg/HgCl_2_ as the reference electrode, and a Pd wire as the counter electrode. The fabricated MXene was used as the working electrode to analyze its electrochemical response. The specific capacitance, energy density, and power density of the fabricated material were calculated as follows:

1 $$C_{sp}=\frac{Area}{2\times \Delta V\times m\times scan\;rate}$$

2 $$C_{sp}=\frac{I\times \Delta t}{\Delta V\times m}$$

3 $$E=\frac{1}{2}CV^{2}$$

4 $$p=\frac{E}{\Delta t}$$

where C_sp_ (F g^–1^) is the specific capacitance, which can be calculated from CV and GCD data; V is the potential window; and the scan rate is expressed in mV s^–1^. I (mA) represent the current at which the GCD was run, t (s) is the discharge time, and m (g) is the loaded mass. E (Wh kg^–1^) is the energy density and P (W kg^–1^) is the power density, which are calculated using eqs 3 and 4.

### 2.5 Mass Balancing for Positive and Negative Electrodes

The mass of the positive and negative electrodes was balanced using the charge-balance relation:

5 $$\frac{m_{+}}{m_{-}}=\frac{C_{-}\Delta V_{-}}{C_{+}\Delta V_{+}}$$

where C_+_ and C_–_ are the specific capacitances of the positive and negative electrodes, and Δ*V*_+_ and Δ*V*_–_ are their respective working voltage windows. The specific capacitance of positive (MXene) and negative (AC) electrodes was calculated using Figure 5b (cyclic voltammetry). The calculated specific capacitance of the positive electrode was 86 F g^–1^ at an operating potential window of 0.5 V, and that of the negative electrode was 22 F g^–1^ at an operating potential window of 0.8 V. Under these conditions, the calculated mass-balance ratio is *m*_+_:*m*_–_ ≈ 1:2.4. This means that if 1 mg of positive material is used, then 2.4 mg of AC should be used to avoid overcharging. All electrochemical measurements were performed using the optimized electrode mass ratio.

The asymmetric device was analyzed through CV measurements at the same scan rate of 30 mV s^–1^, with different potential windows ranging from 0.8 to 1.2 V, as shown in Figure 5c. The CV curves maintained a nearly stable shape without obvious distortion within the 1.2 V range, indicating good electrochemical reversibility and negligible side reactions. The wider operational voltage window contributed to enhanced capacitance and energy density of the device.

Furthermore, the GCD profiles recorded at different potential windows (Figure 5d) at the applied current density of 4 A g^–1^ exhibited nearly symmetric charge–discharge characteristics with a small IR drop (∼0.02 V), confirming the good capacitive behavior and electrochemical stability of the device up to 1.2 V. The selected voltage window was determined based on the stable operating potential ranges of both positive and negative electrodes, while avoiding significant electrolyte decomposition and polarization effects beyond 1.2 V.

### 2.6 Computational Methods (DFT)

Plane-wave DFT calculations were performed using the Quantum ESPRESSO package (version 7.4). The computational workflow was as follows: SCF ground-state calculation → bands.x for band energies → projwfc.x for orbital projections (fat bands and PDOS) → pp.x for real-space charge density. The Fermi level (EF) obtained from the SCF calculations was set to 0 eV for all plots. The Perdew–Burke–Ernzerhof generalized gradient approximation was used with scalar-relativistic pseudopotentials in spin-unpolarized calculations. Electronic smearing based on the Marzari–Vanderbilt method was applied with a width determined by density of states (DOS)-shape convergence criteria. The Ti_3_C_2_T_*x*_ slab included O/F surface terminations, with a vacuum spacing of ≥15 Å along the *z*-direction to prevent spurious interslab interactions. Before electronic property calculations, all structures were fully relaxed according to standard convergence criteria for forces and total energy. Plane-wave and charge-density cutoffs were systematically tested, along with a Γ-centered 2D k-mesh, to ensure convergence of total energy, EF position, and the qualitative features of DOS and band structures. Fat bands were generated by summing orbital-resolved weights from projwfc.x over atoms of the same element and plotting them as marker areas along the k-path. Projected DOS was integrated over selected energy windows (±1 eV for composition analysis; |E| ≤ 3 eV for d-band analysis). The Ti-3d d-band center was calculated as the first moment of the Ti-3d PDOS within an energy window of |E – EF| ≤ 3 eV, covering both occupied and unoccupied Ti-3d states relevant to the proton-coupled surface redox. We note that this descriptor-level definition differs from the conventional Nørskov d-band center, which uses only occupied states; the value reported here should therefore be interpreted as a qualitative descriptor of the proton-coupling tendency rather than a direct binding-energy predictor. Real-space charge density ρ­(r) was obtained using pp.x and visualized as mid-z slices to reveal charge localization patterns.

### 2.7 Antioxidant Assay

MXene antioxidant activity was determined using in vitro antioxidant tests. The radical cation scavenging test known as 2,2-azino-bis­(3-ethyl)­benzothiazoline-6-sulfonic acid (ABTS) was used to evaluate the antioxidant assays. MXene’s ABTS free radical scavenging activity was measured in accordance with the manufacturer’s method (Nanjing Jincheng Bio, China).^27,28^ Various MXene concentrations were added, and the absorbance was recorded at 515 nm. The procedure was performed in triplicate.

### 2.8 Acute Dermal Toxicity

The skin toxicity test was performed in accordance with OECD guideline 402 for the testing of chemicals. This was performed to assess any possible adverse reactions that could occur shortly after a single application of MXene Nanosheet to the skin.^29^ Dermal administration was selected because it represents a potential route of exposure for individuals, allowing us to better understand the associated risks. In summary, approximately 8-week-old male and female SD rats, weighing between 170 and 200 g, were used. Standard animal care conditions were maintained throughout the acute toxicity study. The hydrogel of MXene nanosheets was used at concentrations of 25, 50, and 100 mg/mL/kg, respectively. The hydrogel was applied to approximately 10% of the hairless areas on the backs of male and female rats. Sodium lauryl sulfate, an irritant commonly used in studies, was applied at a concentration of 5%–10% to assess safety of the hydrogel. The areas treated with the hydrogel were covered with specialized bandages that allowed partial air circulation for 24 h. After 24 h, the patches were removed, the application sites were marked, and the skin was washed with water. We observed redness and swelling after removing the patch at 3, 24, and 48 h. Additionally, we monitored the animals for 1 week. The observation included the skin, fur, eyes, respiratory issues (such as drooling, diarrhea, and urination), and effects on the nervous system (such as tremors and seizures, changes in activity level, gait and posture, responses to touch or sound, changes in strength, and unusual or repetitive behaviors). This was followed by a 14-day observation period.^30^ The skin response was assessed using a descriptive grading system at 3, 24, and 48 h after patch removal. Skin reactions were graded using the erythema classification based on the Magnusson–Kligman test.^31^

### 2.9 In Vivo Toxicity Evaluation

#### 2.9.1 Animal Grouping

The subchronic toxicity study utilized male and female SD rats between 8 and 10 weeks old, with body weights ranging from 220 ± 20 g. The rats were kept in polycarbonate cages in a barrier system maintained at a temperature of 20 °C–25 °C, relative humidity of 40%–70%, 12 h light-dark cycle, and room air exchange rate of 10–20 times per h. After dose administration, the feeding density was three rats per cage. Rats were allowed to consume the approved rodent feed and were provided with sterile municipal tap water in water bottles on an ad libitum basis. Each rat was assigned a unique number, and it was necessary to identify them by their ear tag and animal number. Before the trial, none of the rats had undergone any prior surgeries, nor did they have any abnormal clinical conditions.

SD rats were randomly assigned to seven groups (n = 5), including a normal control (NC) group. MXene was administered at varying doses via oral and intraperitoneal routes for 28 days (Figure 10a). All animals were anesthetized with intraperitoneal ketamine (50 mg/kg), and blood samples were collected from the retro-orbital plexus 24 h after the final treatment. Following this, the animals were euthanized by decapitation, their abdomens incised, and the liver, lung, heart, kidney, and spleen were rapidly harvested after irrigation with normal saline. The liver was preserved in 4% formalin for histological examination. The animal studies were approved by the Institutional Committee (Approval No. AE18015).

#### 2.9.2 Serum Biochemistry

To obtain the blood serum, the mouse blood was centrifuged for 15 min at 3500 rpm. The supernatant was collected for analysis. Chemicals manufactured by Nanjing Jincheng Bio, China, were used to quantify the serum levels of blood urea nitrogen (BUN), creatinine (Cre), aspartate aminotransferase (AST), and alanine aminotransferase (ALT).

#### 2.9.3 Enzyme-Linked Immunosorbent Assay (ELISA)

The ELISA kits were used to determine the concentration of inflammatory components in blood serum following the supplier’s instructions. These factors included IL-6 and IL-1β in the blood serum. The ELISA kits for mouse IL-6 and mouse IL-1β were manufactured by Yuanju Bio, Shanghai, China.

#### 2.9.4 Malondialdehyde (MDA) and Glutathione (GSH) Assays

MDA (Nanjing Jincheng Bio, China) and GSH levels were measured in the liver using kits from Solarbio Life Sciences, China, according to the manufacturer’s instructions. This was done to assess the level of oxidative stress caused by MXene nanosheets in the body.

#### 2.9.5 Histological Evaluation

After fixation in 4% neutral formaldehyde solution, the liver, kidney, heart, spleen, and lung tissues were dehydrated using gradient ethanol and then embedded in paraffin. All tissue samples were paraffin-embedded and then sectioned to a thickness of approximately 3 μm. The sections were subsequently stained with hematoxylin-eosin (HE) according to the manufacturer’s instructions. After sealing with neutral glue, the sections were examined under a light microscope at 100× magnification.

### 2.10 Statistical Analysis

All results are presented as mean ± SEM. Unpaired two-tailed Student’s *t* tests or one-way analysis of variance were used to analyze differences between two groups or among multiple groups. Analysis and graphing were performed using GraphPad Prism version 10.1. Statistical significance was defined as *P* < 0.05.

## 3 Results and Discussion

### 3.1 Physiochemical Characterization

Field emission scanning electron microscopy (FE-SEM) was used to perform the morphological analysis of MXene, as shown in Figure 1a–b, with the images showing that the MXene nanosheets are perfectly delaminated. This can be attributed to the Al layer removal, leaving behind well-spaced MXene nanosheets. To support this observation, energy-dispersive spectroscopy (EDS) analysis (Figure 1c–h) was performed, where the intended elements, such as Ti, C, O, F, and Al, were found in desired concentrations. The EDS spectrum shows only X% Al, which is evidence that the Al removal is consistent with the X-ray photoelectron spectroscopy (XPS) and SEM results. Moreover, XRD analysis was performed to verify the MAX phase to MXene conversion. Figure 1i shows the X-ray diffraction (XRD) patterns of MAX and MXene, where the intensity of the Al peak at the (440) plane was reduced to its maximum extent, indicating Al removal. Similarly, the characteristic MXene (002) plane shifted from 9.5 to 9.3, indicating MXene synthesis, as shown in (Figure 1j). Moreover, the (004) and (110) planes are indicative of MXene synthesis.

**Figure 1:**
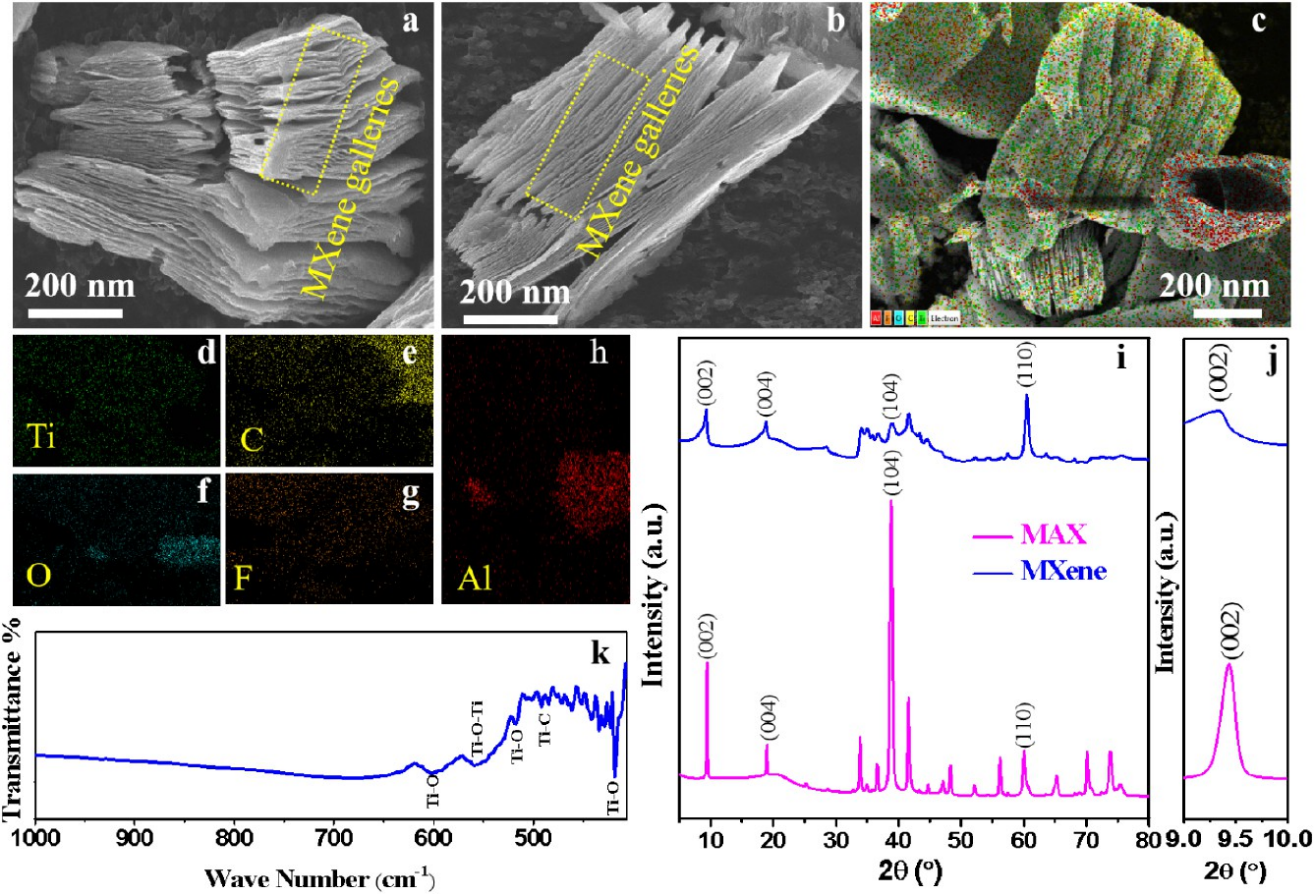
Morphological analysis of MXene using FE-SEM images (a, b), EDS spectrum to visualize element mapping (c), consists of Ti (d), C (e), O (f), F (g), and Al (h). XRD spectrum of MAX phase and MXene (i), comparison of (002) plane shift (j), and FTIR analysis of MXene (k).

Fourier-transform infrared spectroscopy (FTIR) analysis was performed to verify the presence of functional groups on MXene nanosheets (Figure 1k). The MXene FTIR spectrum was found to contain Ti–O peaks in the fingerprint region at 417.7, 516.22, and 599.5 cm^–1,^ respectively. The oxygen-rich functionalities on the MXene surface are highly favorable for energy storage applications, particularly in an acidic environment.

XPS analysis of MXene was performed to elucidate its surface chemistry, as shown in (Figure 2). Initially, the survey spectrum in (Figure 2a) shows all the characteristic peaks of Ti, C, and O. Additionally, the Ti 2p spectrum was further deconvoluted into Ti^3+^ (455 and 461 eV) and Ti^4+^ (456 and 462.1 eV), respectively. Similarly, the O 1s spectrum was deconvoluted (Figure 2b) to identify the key binding energies representing surface functionalities on the MXene surface. The O 1s spectrum (Figure 2c) exhibits peaks at 529.63, 530.5, 531.83, and 532.98 eV, which can be attributed to TiO_2_, C–Ti–O, Ti–O–Ti, and Ti–OH, respectively. These findings indicate the rich functionalities of −O and −OH on the MXene surface, making it a hydrophilic and active energy-storage material. The absence of an Al peak in the spectrum confirmed the Al removal, leaving behind a pure MXene sample. Finally, the XPS spectrum of C 1s was deconvoluted into C–Ti, C–C, and C–H/C–O, corresponding to binding energies of 281.87, 284.47, and 285.14 eV, respectively (Figure 2d). In summary, the XPS results indicate the formation of MXene with rich functionalities of −O and −OH, which are well suited for energy storage applications in acidic electrolyte environments.

**Figure 2:**
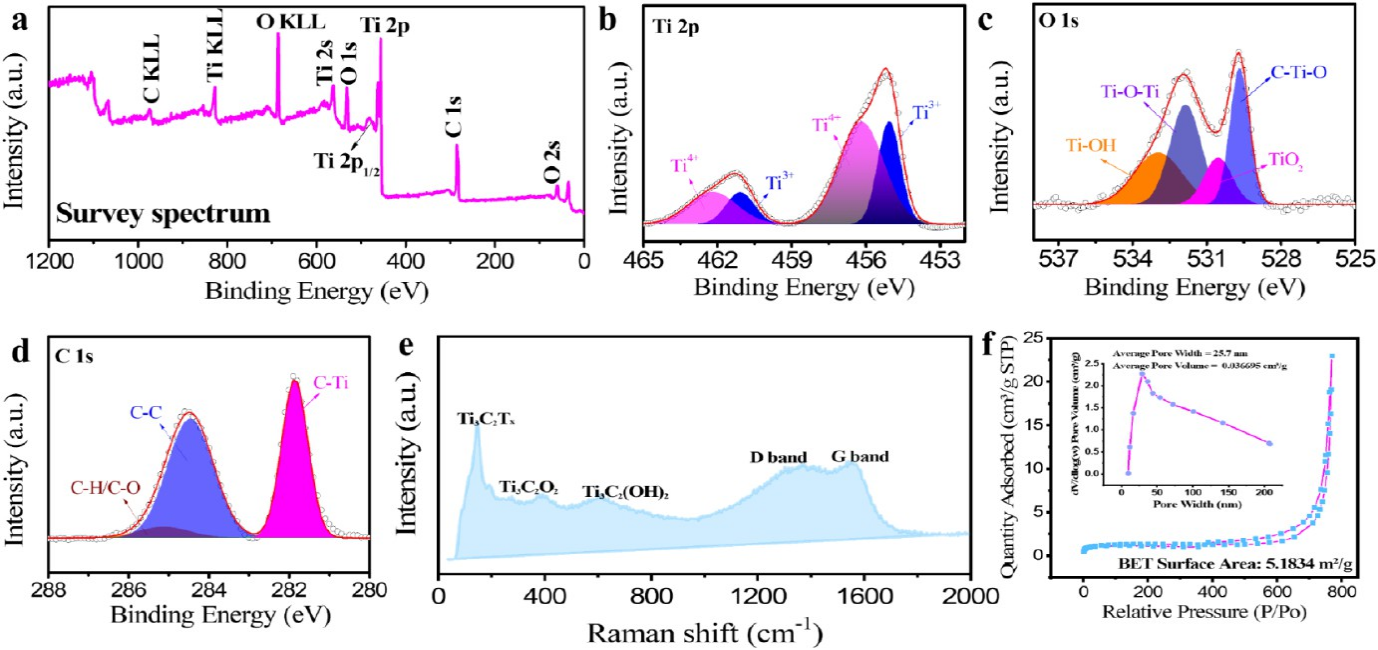
Structural and surface characterization of MXene: XPS survey spectrum (a), high-resolution deconvoluted spectra of Ti 2p (b), O 1s (c), and C 1s (d); surface defect analysis using Raman spectroscopy (e) and BET surface area and pore size distribution analysis (f).

The Raman spectra of MXene (Figure 2e) show characteristic peaks at approximately 145.2, 194.4, 393.3, 515, and 610 cm^–1^. The peak observed at approximately 150–200 cm^–1^ is assigned to Ti_3_C_2_T_*x*_, confirming the MXene structure. Furthermore, the peaks observed near 350–450 cm^–1^ and 600–700 cm^–1^ correspond to Ti_3_C_2_O_2_ and Ti_3_C_2_(OH)_2_, respectively, which are associated with the surface termination groups. The broad peaks at approximately 1350 and 1580 cm^–1^ represent the D and G bands, indicating disordered and graphitic carbon, respectively. These results demonstrate the formation of the layered MXene structure along with −O and −OH rich functionalities and structural disorder, indicating excellent potential for energy storage applications.

Brunauer–Emmett–Teller (BET) analysis (Figure 2f) was performed to evaluate the surface area and pore size distribution on the MXene surface with specific surface area of 5.1834 m^2^ g^–1^, average pore width of 25.7 nm, and pore volume of 0.03665 cm^3^ g^–1^. The adsorption–desorption curve indicates the presence of mesoporous channels, consistent with the layered structure of MXene nanosheets. The pore size distribution confirms that most pores lie within the mesoporous region, which can facilitate electrolyte access throughout the material. The average surface area and pore size distribution provide a readily accessible active surface and enable unhindered diffusion of electrolyte ions during the charge–discharge process.

### 3.2 Electronic Structure Analysis of Ti_3_C_2_T*_x_* MXene

To understand the charge-storage mechanism in Ti_3_C_2_T_*x*_ MXene, we combined DFT electronic-structure analysis with a proton-coupled surface-redox model. (Figure 3a) schematically illustrates the proton-coupled surface redox mechanism, with hydrated H^+^ ions approaching the O/F-terminated Ti_3_C_2_ layers. The proton insertion at the surface terminals initiates a reversible Ti^4+^ ⇄ Ti^3+^ electron transfer while electrons percolate through the metallic Ti–C scaffold. This surface-confined mechanism supports rapid charge-storage kinetics rather than relying on sluggish bulk intercalation, consistent with the observed high-rate pseudocapacitive behavior. (Figure 3b) shows the projection-weighted band structure (fat bands), where the marker area is proportional to the orbital-weight contributions from Ti-3d, O-2p, and C-2p states. Several Ti-3d-rich bands with strong dispersion cross the EF along the k-path that implies low effective mass and high electronic conductivity through the Ti–C framework. This provides the electronic pathway required to sustain rapid proton-coupled surface reactions. (Figure 3c) shows the total and projected DOS, indicating that the states around EF are dominated by Ti-3d orbitals, while O-2p states are predominantly located at lower energies. This electronic structure confirms that the Ti–C scaffold provides delocalized carriers at EF, giving rise to the metallic character, whereas O/F terminations offer chemically active sites for H^+^ adsorption and activation rather than acting as the primary conduction channel. (Figure 3d) quantifies the composition of electronic states within ±1 eV of EF. The integrated contributions are 58.6% for Ti-3d, 36.3% for C-2p, 4.5% for O-2p, and 0.6% for H-1s. Accordingly, approximately 94.9% of near-EF carriers originate from the Ti–C network (Ti-3d + C-2p), while the minor O/H fractions align with localized surface chemistry at the terminations, precisely the division of labor needed for efficient pseudocapacitance. (Figure 3e) shows the Ti-3d partial density of states in |E| ≤ 3 eV, indicating a d-band center located at −0.72 eV relative to EF. The results suggest that MXene possesses reasonable energy storage capabilities (Figure 3f) shows a mid-z slice of the real-space electron density ρ­(r), highlighting regions of high electron density along the interlayer corridor and around surface terminations. This spatial distribution qualitatively aligns with the transport pathways and reaction sites shown in the conceptual mechanism (Figure 3a), confirming the continuous electronic pathway through the scaffold and identifying charge-rich nodes at the terminations where H^+^ insertion occurs. Taken together, these DFT results show that Ti_3_C_2_T_*x*_ has an electronic structure well-suited for pseudocapacitive energy storage: a metallic Ti–C backbone ensures rapid electron transport, O/F terminations enable reversible proton-coupled redox reactions, and the Ti-3d d-band center position (−0.72 eV) supports favorable reaction kinetics. To explicitly correlate the DFT descriptors with the experimental electrochemical response, the electronic-structure features presented in Figure 4 are interpreted together with the kinetic parameters obtained from CV, GCD, EIS, and Dunn analysis (Section 3.3). The Ti-3d and C-2p dominated states near EF (94.9% of near-EF carriers, Figure 4d) indicate a continuous metallic conduction pathway through the Ti–C backbone, which is consistent with the low charge-transfer resistance observed for both the three-electrode MXene cell (Rct = 0.34 Ω, Figure 5d) and the MXene//AC device (Rct = 0.577 Ω, Figure 7f), as well as the small IR drop (∼0.02 V) at 4 A g^–1^ in the GCD profiles. The charge-rich O/F-terminated surface sites identified in the real-space electron density (Figure 4f) provide chemically active regions for proton-coupled redox involving the reversible Ti^4+^ ⇌ Ti^3+^ transition, consistent with the high surface-controlled charge-storage contribution obtained from the Dunn analysis (66.53% at 20 mV s^–1^, increasing to 81.63% at 100 mV s^–1^, Figure 5h), the b value of 0.89, and the CV shapes preserved up to 300 mV s^–1^ in the asymmetric device. The diffusion-controlled contribution that remains at low scan rates (33.47% at 20 mV s^–1^) corresponds to ion access into the interlayer regions of the MXene nanosheets, consistent with the high-density electron channel along the interlayer corridor visible in Figure 4f and with the highest areal capacitance (66.43 mF cm^–2^) observed at the lowest current density (0.5 mA cm^–2^). The moderately downshifted Ti-3d d-band center (−0.72 eV) is qualitatively compatible with reversible Ti valence switching at the surface and with the 95% capacitance retention observed after 5000 GCD cycles. Taken together, the electrochemical behavior of Ti_3_C_2_T*_x_* MXene can be understood as a combined effect of (i) metallic electron transport through the Ti–C backbone, (ii) reversible proton-coupled redox at the O/F-terminated surface, and (iii) limited interlayer ion access that contributes mainly at low scan rates. A summary of the DFT descriptor versus experimental evidence is provided in (Table 1).

**Figure 3:**
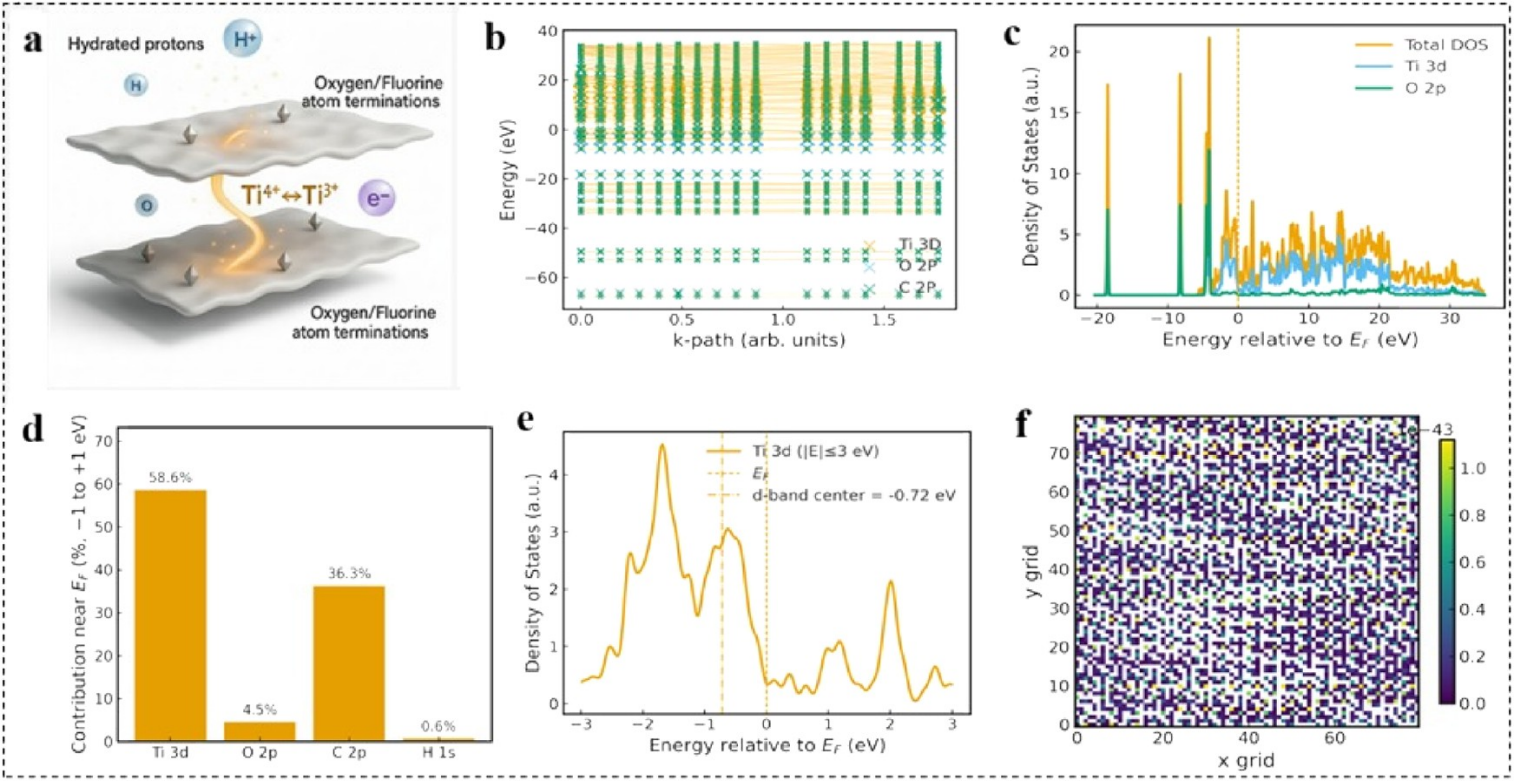
Mechanism and electronic structure underpinning proton-coupled pseudocapacitance in Ti_3_C_2_T*_x_* MXene. Schematic illustration of the proton-coupled redox mechanism, in which hydrated H^+^ inserts at O/F terminations and drives a reversible Ti^4^^+^ ⇌ Ti³^+^ transition while electrons percolate through the metallic Ti–C scaffold (a). Projection-weighted band structure (fat-bands); marker area ∝ orbital weight (Ti-3d, O-2p, C-2p); Ti-3d-rich, dispersive bands cross E_F (b). Total and projected DOS showing Ti-3d dominance at E_F and O-2p states at lower energies (c). Fraction of states within ±1 eV of E_F: Ti-3d 58.6%, C-2p 36.3%, O-2p 4.5%, H-1s 0.6% (d). Ti-3d PDOS within |E| ≤ 3 eV and the corresponding d-band center (−0.72 eV), consistent with reversible proton-coupled Ti valence switching (e). Mid-z slice of the real-space electron density ρ, highlighting high-density regions along the interlayer corridor and around terminations (f). (All plots: boxed axes, no titles, no gridlines; E_F indicated by a vertical dashed line.).

**Figure 4:**
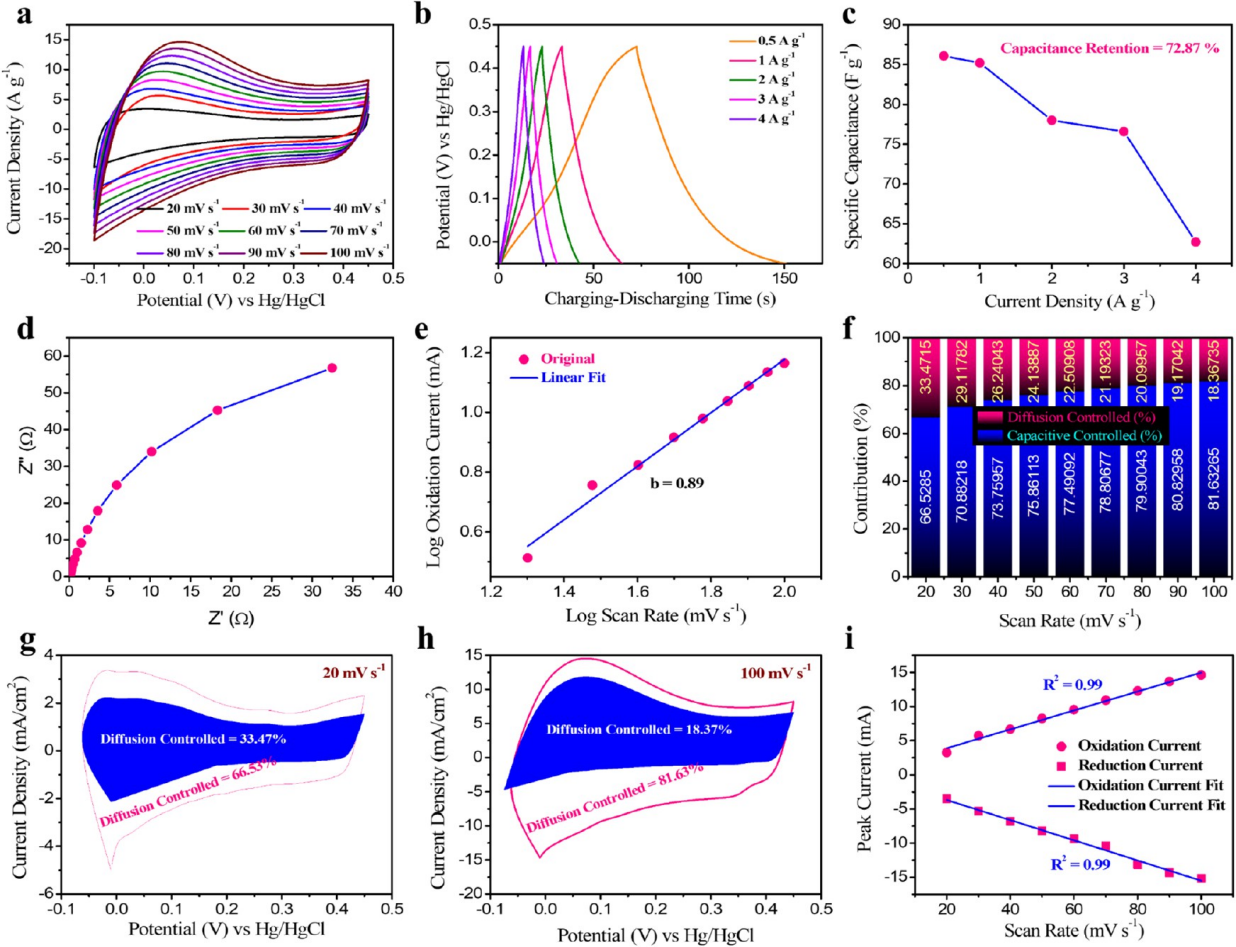
Three-electrode testing of MXene electrode in 3 M H_2_SO_4_, such as CV at the potential sweep ranging from 20 to 100 mV s^–1^ (a), GCD from 0.5 to 4 A/g (b), specific capacitance of the electrode at above current densities (c), and EIS analysis to verify R_ct_ value of MXene electrode (d). Dunn’s method for calculating the “b” value (e) is presented, along with a bar graph illustrating the percentage charge storage mechanism (f), a brief charge storage illustration at 20 mV s^–1^ (g), 100 mV s^–1^ (h), and the anodic and cathodic current regression coefficients (i).

**Figure 5:**
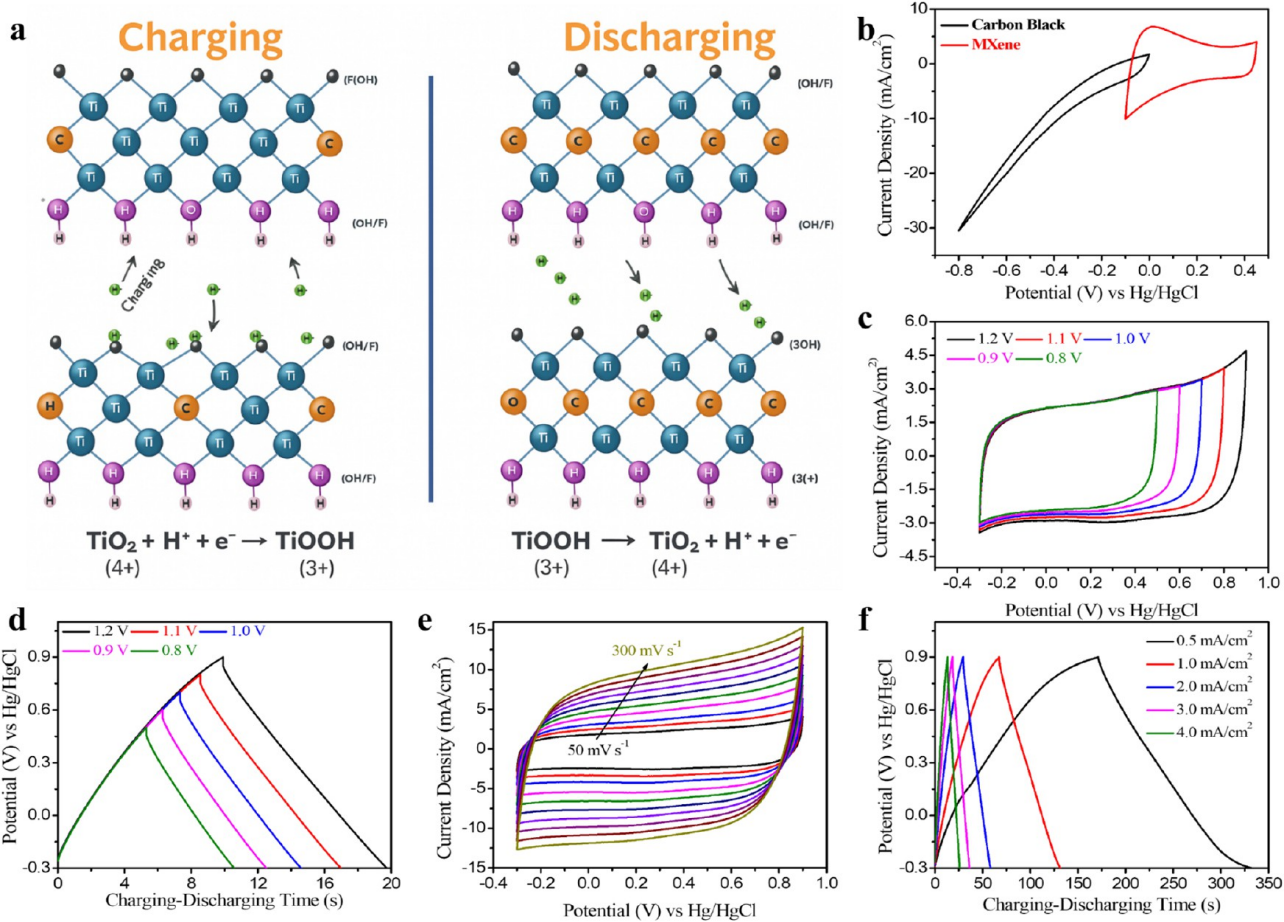
Charging–discharging mechanism of MXene in acidic electrolyte with H^+^ ions interaction with terminal oxygen and proposed reactions during charging and discharging (a). Working potential windows of carbon black and MXene (b), potential window optimization using CV (c), GCD (d), current density from 0.5 to 4 mA/cm^2^ (e) and effect of scan rates ranging from 30 mV s^–1^ to 300 mV s^–1^ (f).

**Table 1:** Correspondence between DFT Descriptors and Experimental Electrochemical Observations for Ti_3_C_2_T*_x_* MXene

DFT descriptor	Value/feature	Experimental observation	Manuscript reference
Ti-3d/C-2p states near EF	94.9% of states within ±1 eV of EF	Low Rct (0.34 Ω three-electrode; 0.577 Ω device); small IR drop (∼0.02 V) at 4 A g^–1^	Figures 3b–d, 4d, 5d, 6f
O/F-terminated surface charge density	Charge-rich nodes at terminations (Figure 3f)	Surface-controlled contribution 66.53 → 81.63% (20 → 100 mV s^–1^); b = 0.89	Figures 3f, 4e–h
Interlayer charge corridor	High electron density along interlayer (Figure 3f)	Diffusion contribution 33.47% at 20 mV s^–1^; 66.43 mF cm^–2^ at 0.5 mA cm^–2^	Figures 3f, 4h, 6a
Ti-3d d-band center	–0.72 eV (moderate H binding)	Reversible Ti^4+^/Ti^3+^ (XPS); 95% capacitance retention after 5000 cycles	Figures 2b, 3e, 6h

### 3.3 Electrochemical Characterization

The electrochemical analysis of the MXene-based electrode was performed to verify its utility in the energy storage system. Primarily, cyclic voltammetry analysis was performed at scan rates ranging from 20 to 100 mV s^–1^, as shown in (Figure 4a). MXene-based electrodes exhibit excellent capacitive current values at the applied potentials, which can be attributed to the surface functionalities of MXene, its 2D conductive sheets, and the Ti–C hybrid structure. Moreover, the fabricated material exhibits an excellent peak shape even at high applied potentials owing to its stable architecture. Similarly, the galvanostatic charging–discharging (GCD) technique was used to visualize the charging–discharging time, which is directly related to the specific capacitance (Figure 4b). The GCD analysis was performed at current densities of 0.5, 1, 2, 3, and 4 A g^–1^. Based on the discharging time, the calculated specific capacitance values are 86.1, 85.1, 78.0, 76.6, and 62.72 F g^–1^ as shown in (Figure 4c). Moreover, the fabricated material retains 72.87% of its capacitance even at 4 A g^–1,^ demonstrating its excellent rate capability. Afterward, electrochemical impedance analysis was performed to determine the charge transfer resistance (R_ct_) at the electrode–electrolyte interface (Figure 4d), and a very small R_ct_ (0.34 ohm) value was observed owing to the conductive MXene architecture. The observed b value of 0.89 indicates that the charge storage on MXene is predominantly surface-controlled (Figure 4e), with a significant pseudocapacitive contribution arising from the O/F-terminated MXene surface. This is consistent with the proton-coupled Ti^4+^/Ti^3+^ redox mechanism identified by the DFT analysis in Section 3.2, and with the dominant role of surface-confined charge storage that becomes increasingly evident as the scan rate is increased. To further support this, Dunn’s method was used, which provides a quantitative measure of the percentage contribution to the charge-storage mechanism at the respective applied scan rates. As shown in (Figure 4f), MXene exhibits approximately 66.52% capacitive charge storage contribution at 20 mV s^–1^ and 33.47% diffusion-controlled (Figure 4g). The figure shows the percentage contributions of diffusion-controlled and capacitive-controlled charge storage processes in the MXene electrode at scan rates ranging from 20 to 100 mV s^–1^. At lower scan rates, diffusion- and capacitive-controlled processes contribute to the overall charge storage. At 20 mV s^–1^, the capacitive contribution is 66.53%, whereas the diffusion-controlled contribution is 33.47%. This indicates that, at slower scan rates, ion diffusion into the inner active sites of the MXene layers still plays a considerable role. At 50 mV s^–1^, the capacitive contribution reached 75.86%, whereas the diffusion-controlled contribution decreased to 24.14%. This trend continued at 100 mV s^–1^, where the capacitive contribution increased to 81.63%, and the diffusion-controlled contribution decreased to 18.37% showed in (Figure 4h). These results indicate that charge storage becomes predominantly surface-controlled at higher scan rates. This behavior is attributed to the high conductivity of MXene and accessibility of its surface-active sites, which promote rapid charge transfer and fast ion adsorption and desorption during electrochemical operation. In contrast, diffusion-controlled processes contribute less at higher scan rates because electrolyte ions have less time to penetrate the deeper active regions of the electrode material. Finally, high R^2^ values were observed for the anodic (0.99) and cathodic (0.99) peak currents at the applied potential (Figure 4i). These high regression coefficients confirm the reproducibility of the fabricated material for energy storage applications.

Based on the present discussion, it is crucial to understand the charging–discharging mechanism of MXene in an acidic environment. This is shown in (Figure 5a), where H^+^ ions from the electrolyte adsorb at the surface −O and −F terminations of MXene, as illustrated by the equation in (Figure 5a). This proton-coupled electron transfer drives a reversible Ti^4+^ ⇌ Ti^3+^ valence switching that is confined to the MXene surface terminations and is distinct from bulk TiO_2_-type redox^+^. MXene’s oxidation state switching is responsible for charge storage (e^–^), which can be used later. On the contrary, during discharging, the H^+^ attached with terminal −O and −F release their respective electrons and move back into the solution. These released electrons can be used as an energy source to power electronics. This can be further supported by the switching of the Ti oxidation state from T^3+^ to Ti^4+^ through electron release. After the three-electrode verification, an asymmetric device was fabricated, consisting of activated carbon as the cathode and MXene as the anode, as presented in (Figure 5b). Activated carbon can operate in the negative potential range, and the activated material in the positive potential range. Subsequently, the asymmetric device was analyzed via CV measurements at a scan rate of 30 mV s^–1^, using potential windows ranging from 0.8 to 1.2 V (Figure 5c). The CV curves retained a nearly stable shape without obvious distortion in the 1.2 V range, which is indicative of good electrochemical reversibility and negligible side reactions. The broader operating voltage window contributed to enhanced capacitance and energy density of the device. Furthermore, the GCD profiles recorded at different potential windows (Figure 5d) at an applied current density of 4 A g^–1^ exhibited nearly symmetric charge–discharge characteristics with a small IR drop (∼0.02 V), confirming the device’s strong capacitive behavior and electrochemical stability up to 1.2 V. The selected voltage window was determined based on the stable operating potential ranges of the anode and cathode, while avoiding considerable electrolyte decomposition and polarization effects beyond 1.2 V. Subsequently, the fabricated device was tested over potential window ranges from 30 to 300 mV/s (Figure 5e). The figure shows that the device exhibits a near-rectangular CV shape, indicative of predominantly surface-controlled charge storage with a significant pseudocapacitive contribution. This shape is attributed to the metallic Ti–C framework of MXene, which provides rapid electron transport, combined with reversible proton-coupled Ti^4+^/Ti^3+^ redox at the O/F surface terminations, as supported by the DFT electronic-structure analysis (Section 3.2). Moreover, the CV shape was retained even at a very high operating potential of 300 mV/s, owing to the mechanical and chemical stability of MXene nanosheets. Additionally, the device’s charging–discharging time was evaluated at varying current densities of 0.5, 1, 2, 3, and 4 mA cm^–2^, with charging–discharging times of 328, 133, 58, 37, and 26 s (Figure 5f). The longest charging and discharging time at the lowest current density can be attributed to shallow and deep electrolyte adsorption, which is typically responsible for high charge storage capability. However, at higher current densities, the shorter charging–discharging time can be attributed only to the adsorption of shallow ions, which results in reduced charging owing to rapid current density switching. Based on the charging–discharging time, the areal capacitance of the fabricated device was calculated (Figure 6a). The fabricated device exhibits the highest capacitance, 66.43 mF cm^–2^, at 0.5 mA cm^–2^, which can be attributed to the penetration of electrolyte ions into the MXene nanosheets. With a regular increase in current density of 4 mA cm^–2^, the areal capacitance decreases to 40.9 mF cm^–2^. The results show 61.61% capacitance retention at high current density, confirming the excellent rate capability of the fabricated device for practical applications. The overall decrease in areal capacitance at high current densities may result from reduced diffusion-controlled charge storage, which can be confirmed using the Dunn real method (Figure 6b).

**Figure 6:**
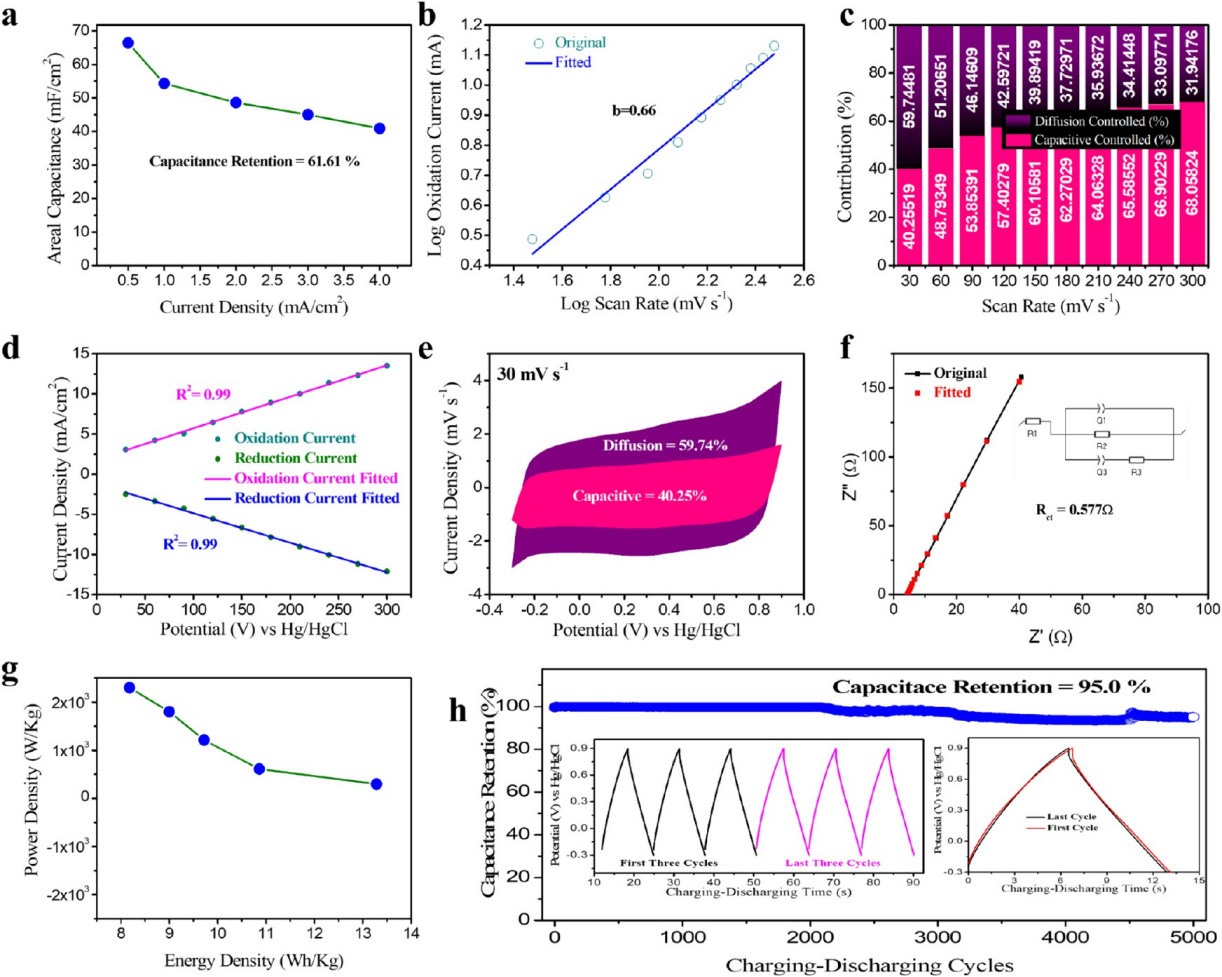
Areal capacitance of asymmetric device at the current densities of 0.5, 1, 2, 3, and 4 mA/cm^2^ (a), calculation of “b” value (b), percentage charge contribution (c), regression coefficient of anodic and cathodic peak current values (d), and detailed illustration of charge contribution at 30 mV s^–1^ (e). EIS analysis of asymmetric device (f), energy density and power density (g), and capacitance retention at 5000 GCD cycles (h).

The calculated “b” value falls within the capacitive-controlled charge storage region (0.66), which can be attributed to the carbon-rich backbone of MXene as the anode and activated carbon as the cathode. The detailed bar graph is shown in (Figure 6c) to illustrate the percentage charging mechanism at applied potentials ranging from 30 to 300 mV s^–1^. At 30 mV s^–1^, diffusion-controlled charging is highest (59.7%) owing to the slow charging process, which allows electrolyte ions to penetrate the MXene nanosheets. However, with the increase in applied potential, diffusion-controlled charging decreases because the rapid potential variation leads only to shallow charging. The contribution of diffusion-controlled charging was only 31.59% at 300 mV s^–1^, which is almost half of that at 30 mV s^–1^. (Figure 6d) shows the excellent reproducibility of the fabricated device, as indicated by the high coefficient values for the cathodic (0.99) and anodic (0.99) peak currents. Finally, a Dunn real graph is shown in (Figure 6e) to illustrate the capacitive and diffusion-controlled areas in the CV at 30 mV s^–1^. These results clearly indicate that the maximum charge storage capacity can be achieved at lower applied current densities owing to shallow and penetrating electrolyte ions. At lower current density, the diffusion-controlled mechanism is dominant, which is responsible for the high capacitance, allowing more charges to be stored through the diffusion of electrolyte ions inside the MXene nanosheets. The EIS discussion includes equivalent circuit fitting, as presented in (Figure 6f). The fitted circuit includes the solution resistance (R1), charge-transfer resistance (R_ct_), and constant phase elements related to the electrode and electrolyte interfaces. The low R_ct_ value of 0.577 Ω indicates rapid charge transfer and good electrical conductivity in the MXene/AC device. Furthermore, the small intercept observed in the high-frequency region reflects low internal resistance, while the nearly linear trend in the low-frequency region indicates efficient ion diffusion and favorable capacitive behavior. These results suggest that the conductive MXene network, along with the porous activated carbon structure, provides effective pathways for rapid electron transport and improved access of electrolyte ions. Similarly, the energy density and power density of the asymmetric device were calculated, as shown in (Figure 6g). The asymmetric device exhibits an excellent energy density of 13.2, 10.86, 9.72, 8.18 Wh kg^–1^ with power densities of 297.07, 611.73, 1215.73, 1801.0, and 2300.6 W kg^–1^ at applied current densities of 0.5, 1, 2, 3, and 4 mA cm^–2^. For a more accurate comparison with the literature, the areal energy density was calculated and found to be 0.132 mWh cm^–2^ at 0.5 mA/cm^2^. The highest energy density and lowest power density at the minimum applied current (0.5 mA) may be attributed to the charge across the entire surface and the diffusion of electrolyte ions inside the MXene nanosheets, which require time for discharging and are responsible for the low power density. However, at the highest applied current density (4 mA cm^–2^), the energy density is minimal, and the power density is at its highest, which can be attributed only to surface-active charge storage, which stores less energy and quickly delivers energy. Finally, the stability analysis of the MXene//AC asymmetric device was performed for 5000 GCD cycles at 6 mA, as shown in (Figure 6h). The device exhibits approximately 95.0% capacitance retention after 5000 GCD cycles, indicating the excellent stability of the MXene nanosheets. Moreover, the inset figures are presented to compare the first and last cycles, providing a clear understanding of capacitance retention. The first and last GCD cycles overlap, which can be attributed to stable MXene performance.

A comparison table (Table 2) has been included to verify that the capacitance, energy density, and cycling stability reported in this study are comparable to those of previously reported MXene-based energy storage devices. This comparison clearly demonstrates the good electrochemical performance of the fabricated MXene-based devices and highlights their potential for further biocompatibility studies.

**Table 2:** Comparison of the Electrochemical Performance of Recently Reported Transition-Metal MXene-Based Supercapacitor Devices, Including Areal Capacitance, Areal Energy Density, and Cycling Stability, with the MXene//AC Asymmetric Supercapacitor Developed in This Work

No.	Electrode Configuration	Electrolyte	Areal Capacitance (mF cm^–2^)	Energy Density	Cycling Stability	Refs
1	Ti_3_C_2_T_*x*_ MXene MSC	Gel electrolyte	39.6	_	85.5% after 12,000 cycles	^32^
2	Ti_3_C_2_T_*x*_//VN-PC ASC	6 M KOH	_	12.81 Wh/kg	73% after 10,000 cycles	^33^
3	Ti_3_C_2_T_*x*_ MXene flexible electrode	PVA/H_2_SO_4_	242	_	90% after 10,000 cycles	^34^
4	Inkjet-printed MXene supercapacitor	GO solid electrolyte	23.0	8.4 μWh cm^–2^	90% after 10,000 cycles	^35^
5	V_2_C MXene supercapacitor	KOH electrolyte	148	52 μWh cm^–2^	87% after 5000 cycles	^36^
6	MXene//AC	PVA-based gel electrolyte	66.43	13.2 Wh/kg or 132 μWh cm^–2^	95% after 5000 cycles	*This Work*

Additionally, the stable performance of the fabricated device across a wide range of bending angles is shown in (Figure 7). Initially, GCD analysis was performed at 0° (Figure 7a) as a reference for comparison with other bending angles. Subsequently, the asymmetric device was fixed at bending angles of 30°, 60°, and 90°, as shown in (Figure 7b–d). GCD analysis was then performed at these bending angles, as shown in (Figure 7e). The figure shows a slight deviation between the charging and discharging times at 30°, while they are nearly identical at 60° and 90°. The slight decrease in charging–discharging time in the bent device can be attributed to the increased resistance at the electrolyte–electrode interface, which reduces the full utilization of active sites. Similarly, the bendable device was attached to the hand and analyzed when the hand was flat and when the hand was bent (Figure 7f–g). The GCD analysis in (Figure 7h) shows nearly identical charging–discharging times for the flat and bent hand, which supports the use of the fabricated device in electronics that can be attached to human bendable joints. Finally, (Figure 7i) shows the components of an asymmetric device consisting of MXene and activated carbon as electrodes, separated by Whatman filter paper to prevent a short circuit.

**Figure 7:**
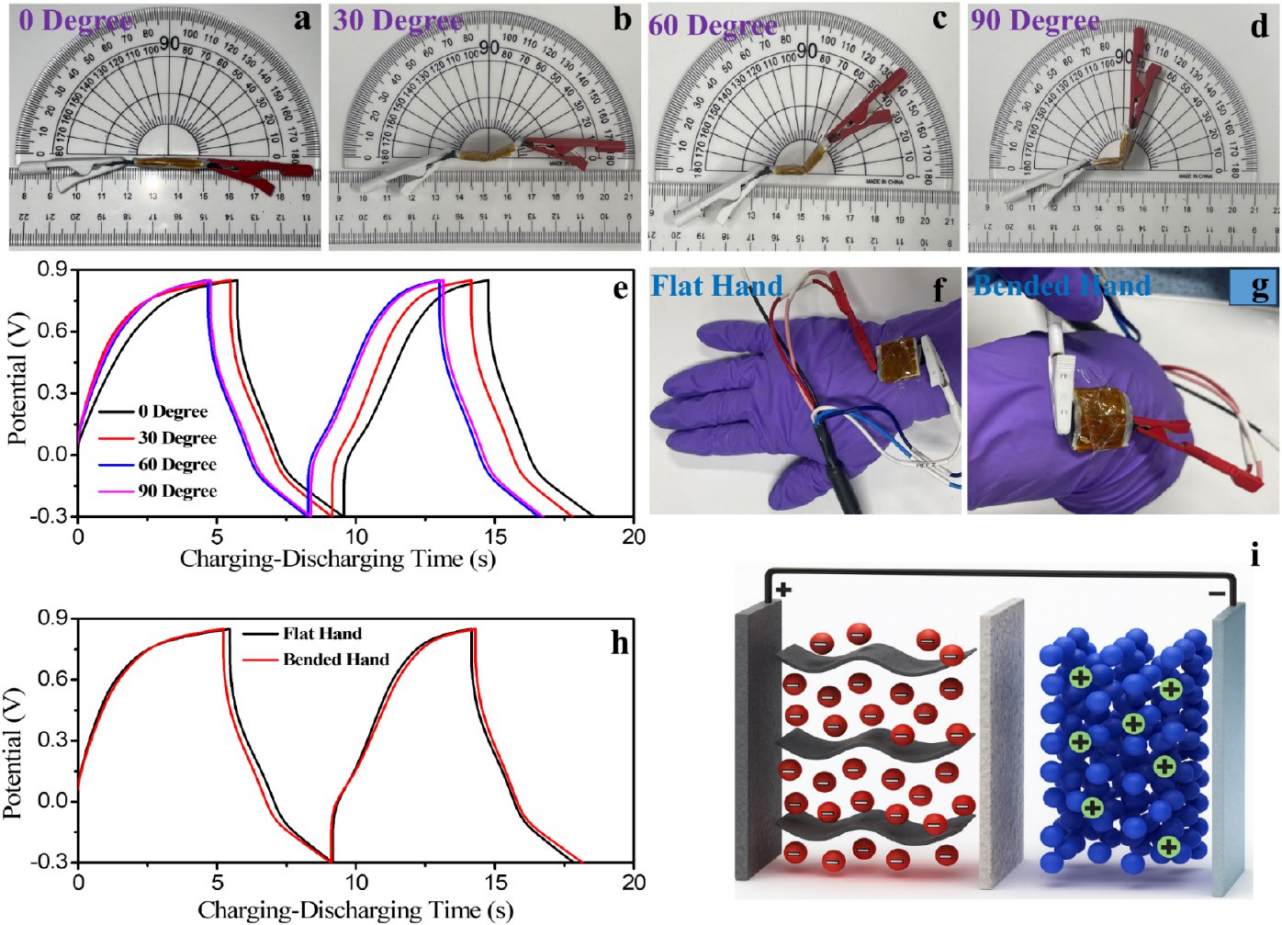
Highly bendable asymmetric supercapacitor device for real-time applications. Bending angles of 0 degree (a). Bending angles of 30 degree (b). Bending angles of 60 degree (c). Bending angles of 90° (d). Comparison of charging and discharging time at these respective angles (e). Asymmetric device at flat hand (f). Asymmetric device at bent hand (g). Charging–discharging time comparison at flat and bent hands (h). Fabrication of an asymmetric supercapacitor device consists of MXene as the positive electrode, activated carbon as the negative electrode, on a carbon cloth substrate (i).

### 3.4 Postcycling Structural and Surface Chemistry Evolution

After 5000 GCD cycles, Raman analysis was conducted and compared with fresh MXene to analyze structural defects before and after cyclic stability on MXene sheets. Raman analysis in (Figure 8a), reveals increased defect density after cycling, supported by the enhanced D-band intensity and the appearance of Ti_3_C_2_O_2_/Ti_3_C_2_(OH)_2_ vibrational features, supporting the formation of oxygenated surface terminations. These observed results indicate that electrochemical cyclic testing is responsible for defect generation and partial oxidation of MXene while preserving the overall layered structure. Additionally, the variation in crystalline structure and interlayer spacing after cyclic testing was analyzed through XRD analysis (Figure 8b). The XRD pattern after cyclic stability shows additional diffraction peaks for Ti_2_O_3_, rutile TiO_2_, and anatase TiO_2_, which can be attributed to partial oxidation of MXene during repeated electrochemical cycling.^37^ The observed (002) plane at 26.4 ° can be attributed to the carbon cloth substrate.^38^ The reduced intensity and broadening of the original MXene peaks indicate structural disorder and defect formation, corresponding to Raman findings. The retention of the (110) reflection indicates that the layered MXene structure remains partially preserved, suggesting surface oxidation rather than complete structural degradation. Additionally, the interlayer spacing of postcyclic MXene increased (12.0 Å) compared to fresh MXene (9.5 Å). The increment in the interlayer spacing after cyclic stability analysis can be attributed to the layers’ swelling due to the diffused electrolyte ions. The observed results indicate the increased structural defects, partial oxidation, and increased interlayer spacing of MXene sheets.

**Figure 8:**
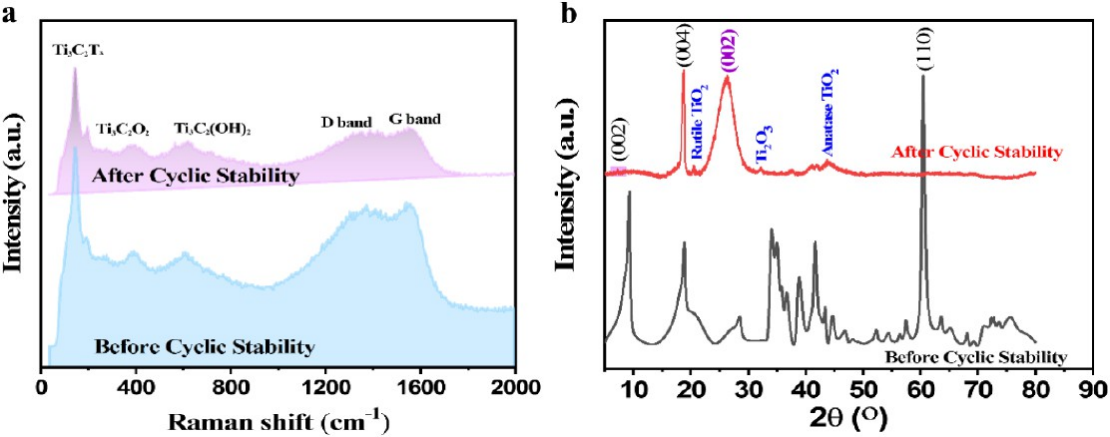
Raman (a) and XRD (b) analysis of MXene before and after cyclic stability testing.

Similarly, the XPS analysis of MXene was conducted after cyclic stability testing to investigate changes in its surface chemistry. (Figure 9a) shows the survey spectrum of MXene, where the intensity of the O 1s peak increased after cyclic stability testing compared to fresh MXene. All other peaks remained similar to those of fresh MXene, indicating oxygen enrichment after cyclic testing with minimal damage to the core structure. To further validate this observation, the Ti 2p peaks were deconvoluted (Figure 9b), and the TiO_2_ (Ti^4+^) content at 456.2 eV (fresh MXene) and 459.0 eV (spent MXene) was compared before and after cyclic testing to analyze the increase in oxygen content after stability analysis. The blue shift of 2.8 eV in the Ti 2p peak can be attributed to the highly electronegative oxygen terminations, which result in higher binding energy.^39^ Furthermore, the calculated TiO_2_ ratio before cyclic testing was 0.28 (area of TiO_2_/total area), while a value of 0.34 was observed after cyclic testing.^40^ The increased TiO_2_ ratio clearly demonstrates the surface oxidation of MXene after cyclic testing. Additionally, (Figure 9c–d) illustrate the C 1s and O 1s spectra, where the presence of C–O and Ti–O related peaks further support surface oxidation. In summary, the postcyclic XPS analysis corresponds well with the XRD and Raman results, confirming the surface oxidation of MXene after cyclic testing.

**Figure 9:**
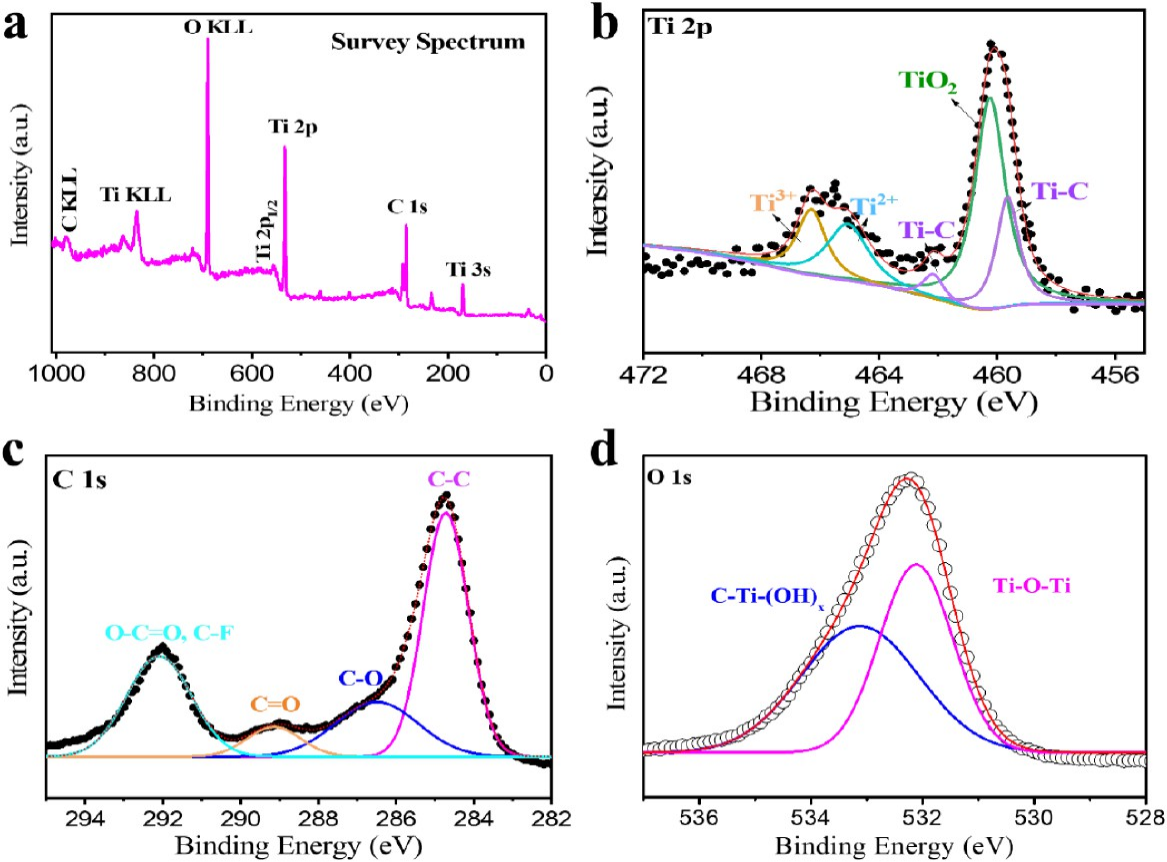
XPS analysis of MXene after cyclic stability testing, including survey spectrum (a) and high-resolution Ti 2p (b), C 1s (c), and O 1s (d) spectra.

### 3.5 Antioxidant Assay

To assess the antioxidant activity of MXene nanosheets, the ABTS radical cation scavenging assay was used. The improved Trolox equivalent antioxidant capacity (TEAC) assay, a well-established method for evaluating antioxidant capacity for over two decades, was used. Unlike the original TEAC assay, which used metmyoglobin/H_2_O_2_ as the radical initiator, the modified version used potassium persulfate (PP) instead. The radical cation used in this assay is ABTS•+ (2,2′-azino-bis­(3-ethylbenzothiazoline-6-sulfonic acid).^41^ The ABTS free radical scavenging activity of MXene is shown in (Figure 10c). The findings showed that MXene effectively inhibited ABTS free radicals at different concentrations, from low to high (500–2000 μg/mL).^42,43^

**Figure 10:**
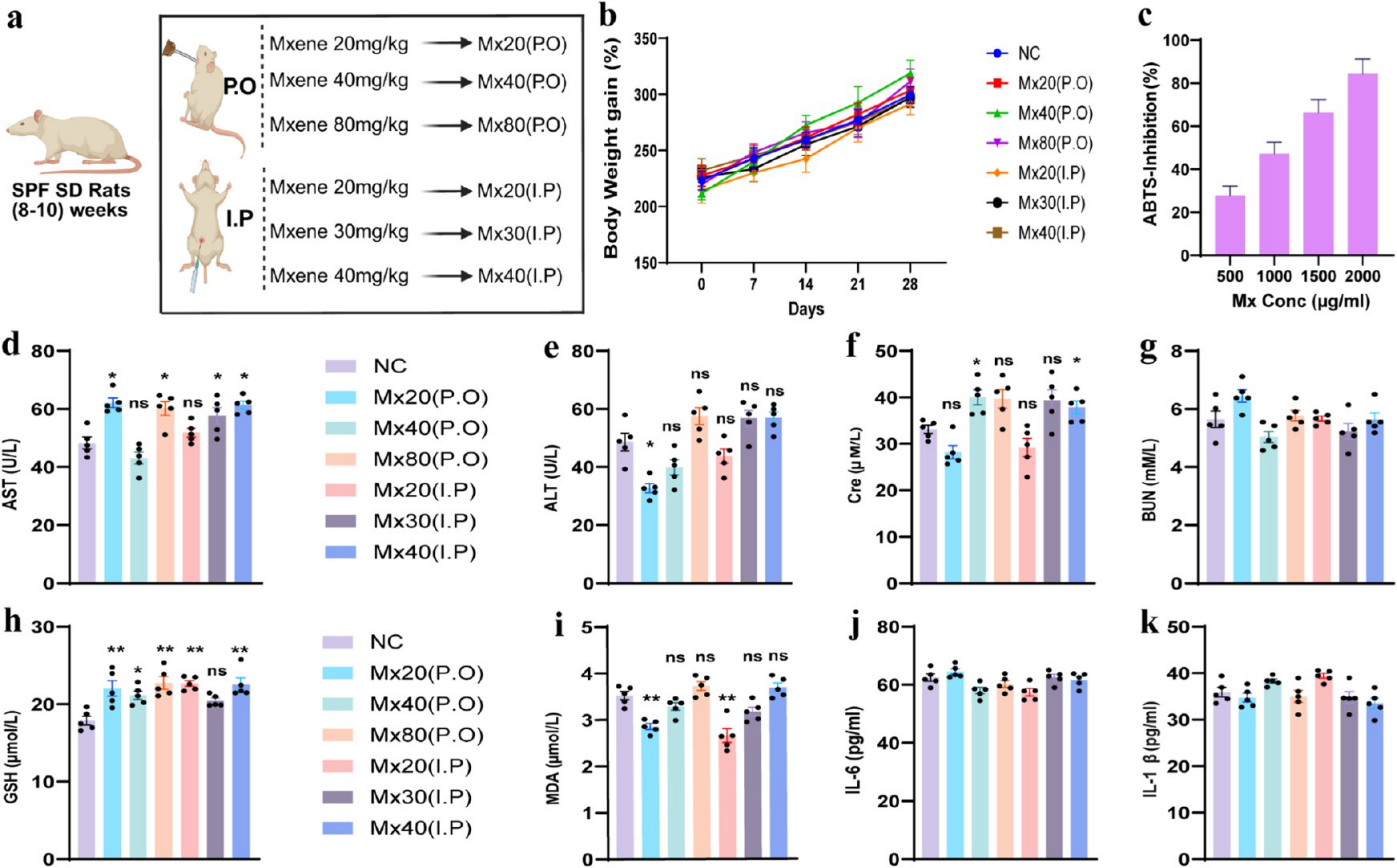
In vivo subchronic toxicity and antioxidant effect of MXene. Grouping of animals for the subchronic toxicity study of MXene by administering orally and intraperitoneally (a). weekly body weight gain of SD rats (*n* = 5) (b). Antioxidant assay using ABTS free radicals (c). Serum levels of AST and ALT in each group (*n* = 5) (d–e). Serum levels of Cre and BUN in each group (*n* = 5) (f–g). The level of GSH and MDA in each group (*n* = 5) (h–i). Serum levels of IL-6 and IL-1β in each group measured by ELISA (*n* = 5) (j–k). All data are presented as the mean ± SD (*P < 0.01, **P < 0.01).

### 3.6 Acute Dermal Toxicity

The study on acute dermal toxicity followed the OECD guideline 402, focusing on potential adverse reactions following single dermal exposure.^29^ Clinical signs were assessed after patch removal at 3, 24, and 48 h for erythema and edema, while body weights were recorded on a weekly basis. According to the Magnusson–Kligman test, (0: no reaction) was observed in the NC (Figure S2) and in MXene 25 and 50 mg/mL, respectively. (1: mild redness, no swelling) was observed in MXene 100 mg/mL at 48 h, and (2: moderate, diffuse redness, no swelling) was observed in the negative control after 3 h. (3: intense redness and swelling) was observed in the negative control after 24 and 48 h. (4: necrosis) was not observed in any of the experimental groups.^31^ The results did not indicate any notable abnormalities or mortality in the rats exposed to MXene nanosheets at various concentrations. Skin reactions were minimal, with no necrosis observed in any of the experimental groups. These findings suggest that MXene nanosheets are relatively safe for dermal applications, even at higher concentrations.

### 3.7 Subchronic Toxicity Evaluation

Toxicity evaluations can be performed on cell cultures (in vitro) and in living organisms (in vivo), including fish, mice, or rats.^30,31^ Various standardized toxicological assays are available to assess the biological response to a chemical compound. However, the absence of standardization in nanoparticle toxicity assessment creates challenges in comparing the toxicity outcomes of the evaluated compounds. Most toxicity assessments for nanomaterials (NMs) have been performed in vitro, using cultures of mammalian cells derived from various anatomical regions, e.g., brain, lungs, heart, skin, and liver.^44^ Despite being less expensive and yielding results faster than in vivo experiments, in vitro data alone cannot be used to draw conclusions about possible health effects in humans.

MXenes are a remarkable class of 2D NMs that are attracting considerable interest in biomedical engineering, particularly in regenerative medicine, infection control, cancer therapy, and biosensing. Moreover, integrating MXenes with other materials considerably enhances their performance, exceeding that of standalone components in medical applications.^3^ This dual modality photothermal/chemotherapy device demonstrated favorable biocompatibility, superior photothermal properties, and a substantial DOX loading capacity. Furthermore, they showed that the drug’s release was triggered by photothermal action, effectively eradicating tumor cells and preventing their recurrence.^45^

#### 3.7.1 Serum Biochemistry

Biochemical parameters are important indices of physiological and pathological status in humans and animals.^46^ Additionally, oxidative stress biomarkers (GSH and MDA) and inflammatory biomarkers (IL-1β and IL-6) are important for investigating drug toxicity because oxidative stress leads to cell death,^47^ and the secretion of inflammatory cytokines causes pathophysiological changes in a healthy body.^48^ The weekly body weight gain is recorded and expressed as the mean ± SEM (Figure 10b), showing the weight gain of SD rats, with no considerable changes compared with the NC. The liver biochemical measure AST exhibited statistically significant variations in the MXene test groups (Mx 30, Mx 80 P.O) and (Mx 40 I.P) relative to the control, whereas ALT significantly decreased only in (Mx 30 P.O) compared with the NC (Figure 10d–e). The liver parameter was within the normal range, indicating that MXene does not have major deleterious effects on liver function at the administered oral and intraperitoneal doses. A marked elevation in Cre levels was noted in the (Mx 40, Mx 80 P.O) and (Mx 30, Mx 40 I.P) groups relative to the NC (Figure 10f). However, Cre levels remained within the normal range, and BUN levels (Figure 10g) showed no changes across all treatment groups compared with the NC, indicating that MXene has a safe profile with respect to renal function at the administered doses.

#### 3.7.2 MDA and GSH Assays

Oxidative stress arises when the levels of oxidants exceed the efficacy of antioxidants in the body, resulting in the production of reactive nitrogen species (RNS). Reactive oxygen species (ROS) produced during oxidative stress interfere with cellular signaling, cause DNA damage, and affect lipids and proteins, ultimately leading to inflammation and apoptotic cell death.^49^ The oxidative stress biomarkers (GSH and MDA) were investigated to measure the oxidative stress exerted by MXene nanosheets. There is a considerable increase in the GSH level (Figure 10h) in the different treatment groups, indicating that MXene has considerable antioxidant potential to scavenge ROS. The MDA in blood serum also showed significant changes in the different treatment groups (Figure 10i) when compared with the control group.

#### 3.7.3 ELISA

Chemicals can stimulate endotoxin production and activate Kupffer cells, leading to the release of inflammatory cytokines, including interleukin (IL)-1β and IL-6. Therefore, these factors can accelerate damage caused by oxidation, inflammatory cell infiltration, and cellular necrosis.^50^ IL-6 and IL-1β levels were quantified via ELISA to investigate the inflammatory mechanism of MXene nanosheets, as shown in (Figure 10j–k). The serum levels of IL-6 and IL-1β did not show notable changes in the MXene treatment groups compared with the NC, confirming that MXene had no effect on inflammatory biomarkers.

#### 3.7.4 Histopathological Analysis

Samples from the liver, spleen, heart, lungs, and kidneys were collected to evaluate treatment-induced histopathological alterations. The tissue samples were preserved in 10% formalin for 24 h. The tissue processing and staining methods were based on Bancroft’s principles and the use of histological techniques.^51^ The histological study of the liver, heart, kidneys, spleen, and lungs revealed no signs of inflammation (Figure 11). Therefore, it is concluded that MXene does not cause any significant histological alterations in the organs under consideration. It is suggested that MXene has a safe profile for vital organs at the tested doses.

**Figure 11:**
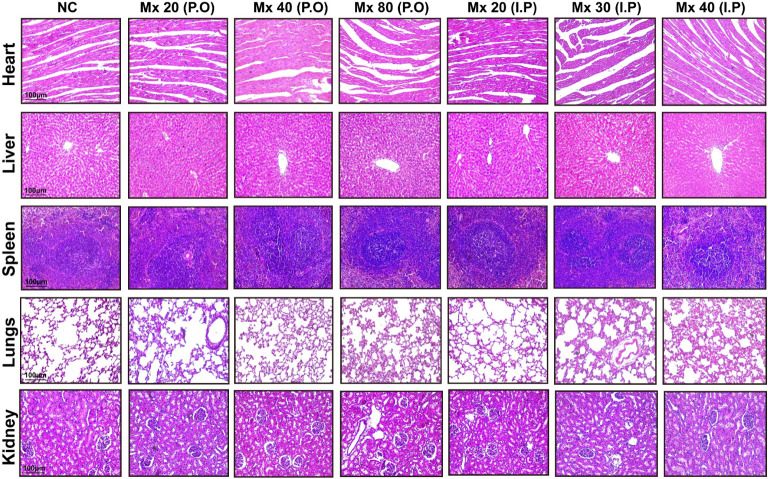
HE staining of different organs, e.g., liver, heart, kidney, lungs, and spleen of the subchronic toxicity study of MXene by administering orally and intraperitoneally (scale bar is 100 μm).

Table 3 provides a comparative assessment of Ti_3_C_2_T_*x*_ MXene with other widely investigated active materials, including graphene/rGO, MnO_2_, MoS_2_, activated carbon, and carbon nanotubes, in terms of relative cost, environmental concerns, toxicity, biocompatibility, and biomedical suitability.^52^

**Table 3:** Ti_3_C_2_T*_x_* MXene Environmental, Cost, and Toxicological Profile of Competing Materials

Materials	Cost	Environmental Concern	Toxicity	Biocompatibility	Biomedical suitability	Ref
MnO_2_	Low	Low to moderate	Neurotoxicity concern	Low	Low	^53^
Graphene	Moderate-High	Toxicant byproducts	Pulmonary inflammation	Very low	Moderate	^7^
MoS_2_ (TMD)	Moderate-High	Moderate	Limited data; genotoxicity suspected	Very low	Low	^54^
Activated Carbon	Very low	Low	Low	Low	Moderate	^55^
Carbon Nanotubes (CNTs)	High	Very low	Lung inflammation and fibrotic effects	Low	Low -moderate	^56^
Ti_3_C_2_T_*x*_ MXene	of MAX phase	Moderate (scalable)	No specific toxicity	Partial	High	this study

To further clarify the biosafety profile of MXene-based materials, the (Table 4) summarizing the previously reported in vivo toxicological studies of Ti_3_C_2_T_*x*_ MXene and related MXene nanostructures has also been included. This table highlights the influence of dose, exposure route, treatment duration, and animal models on the observed biological responses and toxicological outcomes. Overall, both literature reports and our experimental observations suggest that Ti_3_C_2_T_*x*_ MXene exhibits comparatively low organ toxicity and promising biomedical compatibility at experimentally relevant concentrations, although further long-term biosafety investigations are still required.

**Table 4:** Detailed MXene and Related MXene Nanostructures In-Vivo Toxicological Profile

Type of MXene	Dose/Duration	Animal Model	Findings	Refs
Ti_3_C_2_T_*x*_ nanosheets	20 mg/kg (IV) 28 days	ICR mice	Primary distribution in lung and liver; lung accumulation caused respiratory dysfunction, liver accumulation with biliary excretion; no obvious inflammatory lesions	^22^
Nb_2_CT_*x*_ nanosheets	20 mg/kg (IV) 28 days	Kunming mice	Normal hematological parameters; no significant inflammation	^57^
Ti_3_C_2_ quantum dots	10 mg/kg (IV) 14 days	Balb/c mice	No toxic effects; normal blood cell count; no organ histological changes at 1, 7, 14 days	^58^
MnO_*x*_/Ti_3_C_2_–SP	5, 10, 20 mg/kg (IV) 30 days	Mice	Normal vital signs over 30 days; no biochemical or organ toxicity	^59^
Ti_3_C_2_T_*x*_ nanosheets	0.5–2.5 mg/kg (IV) (gestational days)	Pregnant mice	Causes neurological defects in offspring	^60^
Ti_3_C_2_ nanosheets	2.5/5 mg/kg	Mice	Disruption of spermatogenesis	^61^
Au/Ti_3_C_2_T_*x*_	20 mg/kg P.O and IV 14 days	Male/Female wister rats	No evident toxicity.	^62^
Ti_3_C_2_T_*x*_ nanosheets	20, 40, 80 mg/kg (P.O) 20, 30, 40 mg/kg (IP) 28 days	SD rats	No toxicity was seen in all the treatments (ALT, AST, Creatine, BUN) No histomorphological changes were seen in vital organs (Liver, Heart, Kidney, Lungs, Spleen)	This study

Smaller MXene particles, particularly quantum dots (typically < 10 nm), are often preferred for in vivo biological studies owing to their distinct biodistribution and clearance profiles.^63^ Indeed, Ti_3_C_2_T_*x*_ quantum dots have been reported to undergo rapid renal excretion and reduced organ accumulation compared with larger nanosheets, potentially lowering the risk of long-term retention.^64^ Our study deliberately used larger delaminated Ti_3_C_2_T_*x*_ nanosheets lateral size, as shown in (Figure 1a–b) because this form factor is exactly what provides the mechanical flexibility, electrical conductivity, and film integrity required for wearable/implantable supercapacitor electrodes (demonstrated in Figure 6–7). Therefore, assessing the systemic safety of these specific sheets, rather than idealized smaller particles, provides toxicological data that can be directly applied to the intended device application.

After intravenous injection, larger Ti_3_C_2_T_*x*_ flakes (>200 nm) induced minimal inflammatory responses in the mice, whereas smaller fractions (<50 nm) showed higher cellular uptake but also faster clearance.^65^ Importantly, our subchronic oral and intraperitoneal administration of micrometer-scale sheets revealed no significant organ toxicity, histopathological lesions, or oxidative stress at the tested doses, indicating that even relatively large MXene sheets can be biocompatible when appropriately designed. Future work should systematically compare a range of size fractions, from quantum dots to micronscale sheets, under identical administration routes and dosing regimens to establish definitive size–activity relationships for MXene biocompatibility.

The lack of systemic toxicity, inflammatory activation, and histopathological injury after 28 days of multiroute MXene exposure has direct implications for practical device integration. In wearable and implantable bioelectronics, MXene-based electrodes are expected to interface with biological tissue either through direct skin contact (wearable sensors, epidermal electronics) or through subcutaneous/peritoneal implantation (bioelectronic medicine, implantable energy storage). The acute dermal data presented here, showing only mild, reversible erythema at the highest tested dose (100 mg kg^–1^) and no necrosis, directly support the safety of skin-contact wearable configurations, in line with the on-hand bending stability demonstrated in the electrochemical characterization (Figure 7). Likewise, the absence of considerable biochemical disturbances following intraperitoneal administration supports the tolerability of MXene particles that may be released over time from implanted devices into the peritoneal cavity or bloodstream.

However, it is important to interpret these findings in light of the limitations of the current study. While the 28-day subchronic paradigm is compliant with standard regulatory frameworks, it does not capture the potential consequences of the device’s residence over multiple years. Key unresolved questions for long-term integration include cumulative Ti-ion release from device-embedded MXene sheets under physiological redox cycling; fibrotic encapsulation responses at the electrode–tissue interface over chronic time scales; and the potential for MXene nanosheet fragmentation and systemic biodistribution following mechanical fatigue of flexible devices. Furthermore, the dose-dependent ABTS radical scavenging shown by MXene nanosheets indicates antioxidant activity and suggests a potentially beneficial secondary effect in implantable settings, where local ROS generation at the device–tissue interface is a recognized driver of chronic inflammation and device failure. Future studies combining in vivo device implantation with parallel toxicological monitoring will be essential to define the full safety profile of next-generation MXene-based bioelectronic systems.

## 4 Conclusion

This study presents a dual perspective on MXene nanosheets by demonstrating their exceptional electrochemical performance in flexible asymmetric supercapacitors and favorable in vivo safety profile. The fabricated MXene//AC device achieved high areal capacitance (66.43 mF cm^–2^), energy density (13.2 Wh kg^–1^), and power density (2300 W kg^–1^), while retaining 95% of its capacitance after 5000 cycles. Additionally, the device maintained stable output under repeated bending and on-skin attachment. These results highlight the mechanical robustness and reliability of MXene-based devices for wearable and implantable electronics.

In addition to device-level performance, systematic acute dermal and subchronic oral and intraperitoneal toxicity studies in SD rats did not show considerable abnormalities in biochemical markers, oxidative stress parameters, or histopathological assessments of vital organs. Furthermore, MXene nanosheets demonstrated antioxidant activity, supporting their biomedical compatibility. Notably, oral administration was better tolerated than intraperitoneal delivery, in which mild local vacuolization was observed, highlighting the importance of route-specific evaluation for safe biomedical integration.

Overall, the combination of electrochemical efficiency, mechanical flexibility, and favorable in vivo safety presents MXene nanosheets as multifunctional candidates for next-generation implantable bioelectronic systems. Future studies should address long-term safety, potential neurotoxicological effects, and scalable synthesis to accelerate the translation of MXene-based biomaterials into practical medical devices and therapies.

## Supplementary Material



## Notes

Additional information about the instrument/technique used in the characterization of MXene and its acute dermal toxicity study on SD rats (PDF)
