# Supplementary material for: Ti3C2T x MXene Nanosheets: Bridging High-Performance Energy Storage and Comprehensive In Vivo Biocompatibility Assessment
Source: ACS Appl Mater Interfaces. 2026 Jun 15;18(25):34883–901. doi: 10.1021/acsami.6c05337 (PMC13340422; doi:10.1021/acsami.6c05337)
Supplement: Supplementary file 1 [file am6c05337_si_001.pdf]

Supporting Information

$Ti_3C_2T_x$ MXene Nanosheets: Bridging High-Performance Energy Storage and Comprehensive In Vivo Biocompatibility Assessment

Tanveer Ali[a,b1], Ali Shan[c1], Mirza Mahmood Baig[d1]*, He Xu[a], Zhihao Zhao[a], Hou Yuexue[a], Sooman Lim[c]*, Seung Goo Lee[d]*, Lin Zhang[a]*

[a]*Institute of Integrative Medicine, Dalian Medical University, Dalian 0411, China*

[b]*Faculty of Eastern Medicine and Natural Sciences, Ziauddin University, Karachi 74600, Pakistan*

[c]*Graduate School of Flexible and Printable Electronics, LANL, JBNU Engineering Institute, Jeonbuk National University, Republic of Korea*

[d]*Department of Chemistry, University of Ulsan, Ulsan 44610, Republic of Korea*

[a]*Corresponding Author email: zhl8247@163.com

[c]*Corresponding Author email: smlim@jbnu.ac.kr

[d]*Corresponding Author email: lees9@ulsan.ac.kr

[1] Authors equally contributed

Material characterization

Structural and morphological characteristics of the synthesized materials were analyzed using a suite of complementary techniques. The crystalline phase was identified through X-ray diffraction (XRD) using a Rigaku ULTIMA IV diffractometer equipped with Cu Kα radiation (λ = 0.15406 nm or 1.5406 Å), scanned over a 2θ range of 5°–80°. Surface morphology and elemental composition were examined via field-emission scanning electron microscopy (FE-SEM, JEOL JSM-7610F) coupled with energy-dispersive X-ray spectroscopy (EDX). The oxidation states and surface chemical environments of constituent elements were determined using K-alpha X-ray photoelectron spectroscopy (XPS, Thermo Scientific). All FE-SEM and EDX measurements were performed at the Total-Period Analysis Center (TACU), Ulsan Chemical Industry, and Korea Basic Science Institute. Additionally, molecular vibrational signatures were recorded using a Fourier-transform infrared (FTIR) spectrometer (Shimadzu IRAffinity-1).

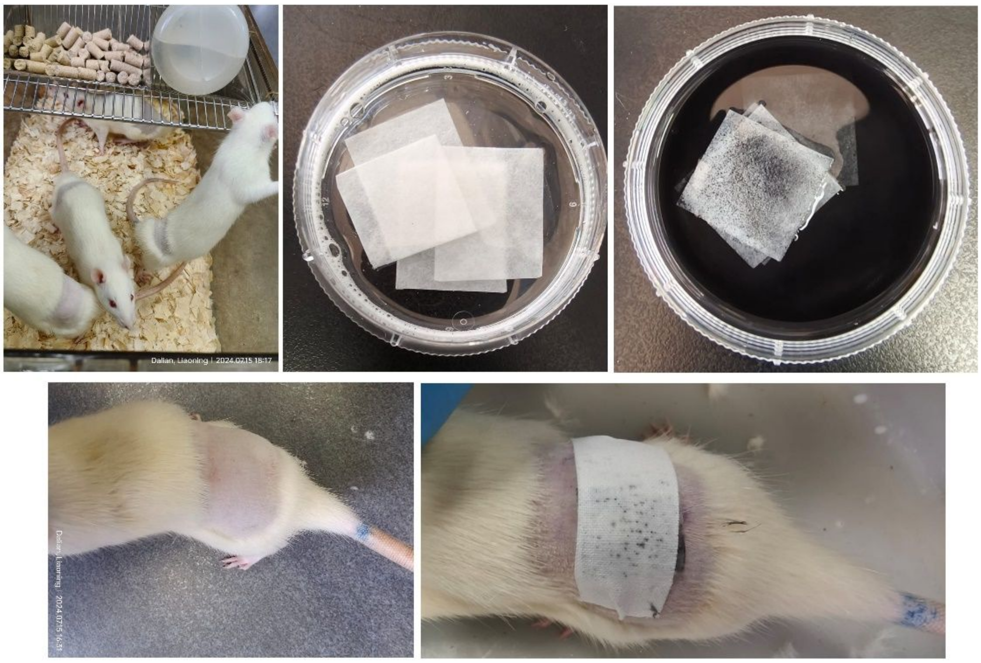

Figure S1: Some Photos during in vivo experiments

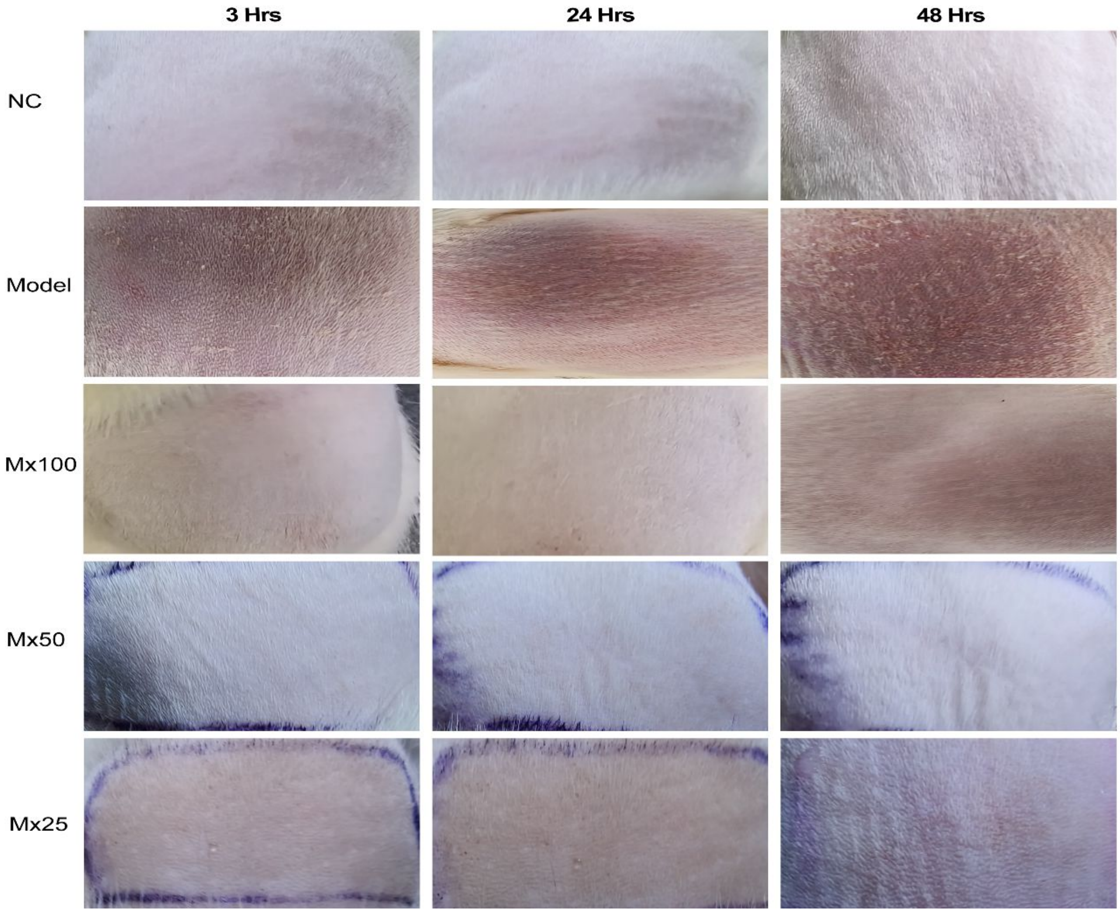

Figure S2. The Acute dermal toxicity study of MXene nanosheet by applying different concentrations.
